# Immunogenicity and Safety of an Inactivated SARS-CoV-2 Vaccine: Preclinical Studies

**DOI:** 10.3390/vaccines9030214

**Published:** 2021-03-03

**Authors:** Ahmed Kandeil, Ahmed Mostafa, Rehab R. Hegazy, Rabeh El-Shesheny, Ahmed El Taweel, Mokhtar R. Gomaa, Mahmoud Shehata, Marawan A. Elbaset, Ahmed E. Kayed, Sara H. Mahmoud, Yassmin Moatasim, Omnia Kutkat, Noha N. Yassen, Marwa E. Shabana, Mohamed GabAllah, Mina Nabil Kamel, Noura M. Abo Shama, Mohamed El Sayes, Amira N. Ahmed, Zahraa S. Elalfy, Bassim MSA Mohamed, Safa N. Abd El-Fattah, Hazem Mohamed El Hariri, Mona Abdel Kader, Osama Azmy, Ghazi Kayali, Mohamed A. Ali

**Affiliations:** 1 Center of Scientific Excellence for Influenza Virus, Environmental Research Division, National Research Centre, Giza 12622, Egypt; Ahmed.Kandeil@human-link.org (A.K.); ahmed_nrc2000@hotmail.com (A.M.); rabeh.elshesheny@stjude.org (R.E.-S.); Ahmed.Nageh@human-link.org (A.E.T.); Mokhtar.Rizk@human-link.org (M.R.G.); Mahmoud.Shehata@human-link.org (M.S.); Ahmed.Elsayed@human-link.org (A.E.K.); Sarah.Hussein@human-link.org (S.H.M.); Yasmin.Moatasim@human-link.org (Y.M.); Omnia.Abdelaziz@human-link.org (O.K.); gaballah09@gmail.com (M.G.); minanabil56@yahoo.com (M.N.K.); noura.mahrous1995@gmail.com (N.M.A.S.); mohameddiaaelsayes@outlook.com (M.E.S.); 2 Department of Pharmacology, Medical Division, National Research Centre, Cairo, Giza 12622, Egypt; rehab_hegazy@hotmail.com (R.R.H.); dr.marawan@gmail.com (M.A.E.); bassim.mohamed@umontreal.ca (B.M.M.); 3 Department of Pathology, Medical Division, National Research Centre, Cairo, Giza 12622, Egypt; nn.yassin@nrc.sci.eg (N.N.Y.); me.shabana@nrc.sci.eg (M.E.S.); zs.el-alfy@nrc.sci.eg (Z.S.E.); 4 Department of Clinical & Chemical Pathology, Medical Division, National Research Centre, Cairo, Giza 12622, Egypt; amira.koraitim@yahoo.com (A.N.A.); sn.abdelfattah@nrc.sci.eg (S.N.A.E.-F.); dr.monaabdelkader@hotmail.com (M.A.K.); 5 Department of Community Medicine, Medical Division, National Research Centre, Cairo, Giza 12622, Egypt; dochzm@gmail.com; 6 Reproductive Health Department, Medical Research Division, National Research Centre, Giza 12622, Egypt; osamaazmy@nrc.sci.eg; 7 Department of Epidemiology, Human Genetics, and Environmental Sciences, University of Texas, Houston, TX 77030, USA; 8 Human Link, Dubai, United Arab Emirates

**Keywords:** SARS-CoV-2, pre-clinical study, vaccine safety, immunogenicity, efficacy

## Abstract

Since the emergence of SARS-CoV-2 at the end of 2019, 64 candidate vaccines are in clinical development and 173 are in the pre-clinical phase. Five types of vaccines are currently approved for emergency use in many countries (Inactivated, Sinopharm; Viral-vector, Astrazeneca, and Gamaleya Research Institute; mRNA, Moderna, and BioNTech/Pfizer). The main challenge in this pandemic was the availability to produce an effective vaccine to be distributed to the world’s population in a short time. Herein, we developed a whole virus NRC-VACC-01 inactivated candidate SARS-CoV-2 vaccine and tested its safety and immunogenicity in laboratory animals. In the preclinical studies, we used four experimental animals (mice, rats, guinea pigs, and hamsters). Antibodies were detected as of week three post vaccination and continued up to week ten in the four experimental models. Safety evaluation of NRC-VACC-01 inactivated candidate vaccine in rats revealed that the vaccine was highly tolerable. By studying the effect of booster dose in the immunological profile of vaccinated mice, we observed an increase in neutralizing antibody titers after the booster shot, thus a booster dose was highly recommended after week three or four. Challenge infection of hamsters showed that the vaccinated group had lower morbidity and shedding than the control group. A phase I clinical trial will be performed to assess safety in human subjects.

## 1. Introduction

Coronaviruses (CoVs) are enveloped viruses with positive-sense, single-stranded RNA genome of non-segmented nature and are divided according to phylogenetic analysis into four genera (Alpha-, Beta-, Gamma-, and Delta-CoVs) within the family Coronaviridae [1]. For many years, two Alphacoronaviruses (HCoV-229E and HCoV-NL63) and two Betacoronaviruses (HCoV-OC43 and HCoV-HKU1) were known to be associated with a mild and self-limited respiratory infection in humans, namely the common cold. This list of human coronaviruses was recently expanded by the addition of three highly pathogenic human Betacoronaviruses, the Severe Acute Respiratory Syndrome Coronavirus (SARS-CoV) in 2003, the Middle East Respiratory Syndrome Coronavirus (MERS-CoV) in 2012, and the Severe Acute Respiratory Syndrome Coronavirus 2 (SARS-CoV-2) in 2019.

Development of different forms of vaccines based on several approaches against SARS-CoV-2 is urgently needed to protect the general population, in particular high-risk groups such as the elderly and healthcare personnel. Several vaccine platforms have been developed or are under development, including recombinant protein vaccine, whole inactivated vaccine, viral vector-based vaccine, DNA vaccine, and mRNA vaccine. Whole inactivated vaccines are the most commonly used types for human and veterinary vaccines. They are relatively easy and rapid to produce. The whole inactivated vaccine production process includes growing the seed virus to an appropriate titer followed by chemical or physical inactivation. The inactivation process leads to loss of infectivity, but retains the main virus antigenicity.

Inactivated SARS-CoV-2 vaccines are produced by growing the virus in cell culture, usually on Vero-E6 cells, followed by chemical inactivation of the virus. They can be produced relatively easily; however, their yield could be limited by the productivity of the virus in cell culture and the requirement for production facilities at biosafety level 3. Examples of inactivated vaccine candidates include CoronaVac (initially known as PiCoVacc), which is developed by Sinovac Biotech in China [2,3], as well as several other candidates that are being developed in China, in India by Bharat Biotech, and in Kazakhstan by the Research Institute for Biological Safety Problems. These vaccines are usually administered intramuscularly and can contain alum (aluminum hydroxide) or other adjuvants. Because the whole virus is presented to the immune system, immune responses are likely to target not only the spike protein (S) of SARS-CoV-2 but also other viral proteins including the matrix (M), envelope (E), and nucleoprotein (N). A previous study showed that the relative quantity of the whole S, cleaved S1/S2, N, M, and accessory 3a protein in the whole inactivated virions was 22.6, 10.6, 51.3, 8.9, and 2.5%, respectively [4]. Due to this variation, the role of each viral protein needs to be further clarified in detail.

An animal model is critical for studying the vaccine efficacy and evaluation of vaccine potency. An ideal experimental animal model of SARS-CoV-2 is that one mimics a human during viral infection and development of viral diseases aspects. Mice, rats, hamsters, guinea pigs, and ferrets were used in previous studies to evaluate several platforms of vaccines in preclinical trials [2,3,4]. Ferrets and hamsters are among the best small experimental animal models, though limited clinical illness and no mortality are observed post viral infection.

Several inactivated vaccine candidates have entered clinical trials, with three candidates from China in phase III trials, one from India, one from Kazakhstan, and two from China in phase I or II clinical trials. Here, we describe the preclinical studies of the first Egyptian inactivated SARS-CoV-2 vaccine candidate (NRC-VACC-01) and illustrate its immunogenicity in hamsters, mice, rats, and guinea pigs, safety using the rat model, and efficacy using the hamster model in preclinical studies.

## 2. Materials and Methods

### 2.1. Vaccine Seed Strain and Cells

SARS-CoV-2 strain hCoV-19/Egypt/NRC-03/2020 (GISAID accession number: EPI_ISL_430819) was isolated in Vero-E6 cells (ATCC No. CRL-1586) from an oropharyngeal swab specimen collected from a 34-year-old Egyptian woman on 18 March 2020. The virus was plaque purified and passaged twice in Vero-E6 cells to generate P2 virus stock in the presence and absence of L-1-tosylamido-2-phenylethyl chloromethyl ketone (TPCK)-treated trypsin. P2 harvests of trypsinized and non-trypsinized viruses were subjected to virial RNA copy number quantification and next generation full genome sequencing to determine virus passaging effects on viral genome stability and its titers.

### 2.2. Virus Propagation, Titration, and Antigen Preparation

P2 virus stock was propagated in Vero-E6 cells cultured in a Nunc cell factory system (Thermo Fisher Scientific, Waltham, MA, USA) at multiplicity of infection (MOI) 0.005, then cells were microscopically investigated daily. The virus-infected culture supernatant was clarified by centrifugation at 4000 rpm for 15 min at 4 °C twice. The harvested virus was titrated by plaque titration assay [5]. Harvested SARS-CoV-2 was inactivated with 0.1% β-Propiolactone (Invitrogen), then the treated virus was incubated at 4 °C for two days in a cooling shaking incubator. The β-Propiolactone treated virus was tested for loss of its infectivity by inoculating it in Vero-E6 cells for seven days. No cytopathic effect (CPE) was observed on cells infected with inactivated virus. Virus harvest (1000 mL) was concentrated by ultra-filtration system (Amersham Bioscience, Amersham, UK) hollow fiber cartridge of 50 KDa pore size and a circulation rate of 150 rpm. The concentrated virus was further concentrated by ultracentrifugation (80,000× *g*, 4 °C, 1 h with 20% sucrose as a cushion). Total protein content was measured by Pierce™ BCA Protein Assay Kit (Thermo Fisher Scientific).

### 2.3. Analysis of Viral Antigen

The concentrated viral antigen was compared with un-concentrated virus using sodium dodecyl sulfate polyacrylamide gel electrophoresis (SDS PAGE) and Western blotting (WB) analysis using specific monoclonal antibodies directed against the nucleoprotein and the receptor binding domain (Sino Biological, Beijing, China). The concentrated viral antigen was fixed using 1% glutaraldehyde, then adsorbed on a carbon-coated, 200-mesh copper grid. The virions were subjected to negative staining followed by examination by transmission electron microscope (JEM-2100 Electron Microscope).

### 2.4. Preparation of NRC-VACC-01 Inactivated Candidate Vaccine

The desired amount of viral antigen (3, 6, 10, 15, 20, 30, 50, 100 µg) was diluted in 1X PBS, then mixed with Imject Alum adjuvant (Invitrogen) in a ratio of 2:1 (*v*/*v*). The formulated NRC-VACC-01 adjuvanted inactivated vaccine was mixed for 30 min under cooling conditions to confirm adsorption of viral antigen into the surface of the alum.

### 2.5. Immunogenicity Studies of the Candidate Vaccine

Four different animal models were used for the evaluation of immunogenicity of the NRC-VACC-01 inactivated candidate vaccine including BALB/c mice (60 females), Albino Wistar rats (60 males and 60 females), Syrian hamsters (60 females), and guinea pigs (60 females). All animals (6- to 8-week-old) were obtained from the animal house colony of the National Research Centre (NRC), Egypt. The animals were verified to be seronegative. The animals were maintained at a controlled temperature of 24 ± 1 °C with a 12–12 h light–dark cycle (light cycle, 07:00–19:00), and were allowed free access to water and standard chow ad libitum. Animals were allocated into five groups (*n* = 12). BALB/c mice, hamsters, and male and female rats were intramuscularly (IM) immunized with 300 μL of vaccine containing 3, 6, 15, and 30 µg of NRC-VACC-01 inactivated virus. Guinea pigs were injected intramuscularly with different doses (300 μL containing 10, 20, 50, and 100 µg/dose) of NRC-VACC-01. Twelve animals of each species were mock-immunized using PBS. The animals were then followed up for ten weeks post vaccination (wpv), and any mortality was recorded. Serum samples were collected weekly from immunized animals till 10 wpv. All animal sera were separated and stored at −20 °C until used.

### 2.6. Viral Microneutralization Assay

Viral microneutralization assay (VMN) was performed to determine the immunogenicity of NRC-VACC-01 against SARS-CoV-2 in the collected sera from different vaccinated and control animal models as previously described [5]. Briefly, serial 2-fold dilutions of heat-inactivated serum samples collected from all animals starting from a dilution of 1:10 to 1:1280 were mixed with equal volumes of 100 TCID 50/mL of hCoV-19/Egypt/NRC-03/2020 SARS-CoV-2 isolate. The mixture was incubated at 37 °C for 1 h, then added in duplicate to cultured Vero-E6 cells in 96-well plates, and incubated for 1 h. The inoculums were aspirated and infection DMEM medium with 2% bovine serum albumin (BSA) was added. The plates were then incubated for three days at 37 °C in 5% CO_2_ in a humidified incubator. The highest serum dilution that completely neutralized the virus was recorded as the neutralizing antibody titer. Seronegative sera were given a value of 1:5.

### 2.7. Detection of Total Specific Antibodies in Rat Sera Using ELISA

To determine the IgG response of different groups of vaccinated and control rats, ELISA was conducted on collected serum samples at 4, 6, 8, and 10 wpv. The 96-well polyvinyl microtiter ELISA plates were coated with 1 μg/mL (100 μL/well) whole inactivated SARS-CoV-2 antigen in 1X Coating Solution (KPL), then incubated at 4 °C overnight. Each coated well was blocked with 100 μL PBST-1% BSA then incubated at 37 °C for 2 h. After three washes with 100 μL PBST washing buffer for each well, plates were loaded with 1:50 diluted rat sera in 100 µL/well blocking buffer and plates were incubated at 37 °C for 2 h. After washing, wells were loaded with 100 µL/well of diluted (1:3000) peroxidase-conjugated anti-rat-IgG (KPL) and incubated at 37 °C for 2 h followed by three washes with washing buffer. For color development, 100 μL/well of OPD substrate (Sigma Aldrich, Missouri, USA) diluted in substrate buffer were used and plates were left for 10 min at room temperature till color development. The enzymatic reaction was stopped using 100 μL 4 M H_2_SO_4_ and the changes in optical density (OD) were recorded at λ max 490 nm using a multi-well plate reader (Biochrom, Cambridge, UK).

### 2.8. Challenge Infection of Hamsters

Ten weeks post vaccination, 14 vaccinated hamsters (seven from the group that received a 6 μg/dose and seven from the 15 μg/dose group) and a control group were individually anesthetized using ketamine–xylazine (K, 100 mg/kg; X, 10 mg/kg). Hamsters were challenged intranasally with 100 µL containing 5.5 × 10^4^ PFU of SARS-CoV-2 (50 µL in each nare) and monitored for 14 additional days [3]. Body weight and temperature were assessed daily. Any mortality or morbidity changes were recorded. On 3, 5, and 7 days post infection (dpi), a subset of animals (3/group) was euthanized using carbon dioxide. Nasal washes and organs (lung, kidney, heart, brain, liver, intestine) were collected from euthanized animals. Organs were divided into two parts. The first part was fixed in 10% formaldehyde, washed, dehydrated, and embedded in paraffin blocks for histopathology examinations. The second part was subjected to homogenization to determine viral titers in each collected organ. Briefly, 0.1 gm of each organ was homogenized in 0.5 mL PBS with a Qiagen Tissue Lyser II (Qiagen, Hilden, Germany). Organ homogenates were centrifuged at 2000× *g* for 5 min. The virus titer was determined in the supernatants of homogenized organs and nasal washes by TCID50/35 µL and plaque titration assay.

### 2.9. Safety Evaluation of NRC-VACC-01 Inactivated Candidate Vaccine in Rats

The baseline weight of each vaccinated rat was recorded. The change of the body weight post vaccination was assessed weekly until the end of the experiment, while changes in food intake were recorded daily. The injection site was daily analyzed for skin abnormalities, as well as the severity of the skin reaction that was categorized and scored as none (0), mild (1), moderate (2), or marked (3) based on the reaction diameter. Only in case of reaction observation, a further necropsy was applied for animals from each group with the most visibly severe reactions at the conclusion of the first three days.

### 2.10. Blood Analysis

On day 3 post vaccination (acute post-immunization stage), 2 wpv (post-immunization short-term phase) and 8 wpv (prolonged-effects phase) blood samples were collected from the tail vein from six female and six male rats/group under intraperitoneal anesthesia with ketamine–xylazine (K, 100 mg/kg; X, 10 mg/kg) [6]. Blood samples were collected in sterile ethylenediaminetetraacetic acid (EDTA) vacutainer tubes for analysis of complete blood counts (CBC) using an automated cell counter (Medonic M20 cell counter), and estimation of plasma D-dimer using a specific ELISA kit (SunLong Biotech, Zhejiang, China). On the other hand, blood samples collected in heparinized tubes were used for determination of SGOT, SGPT, albumin, urea, creatinine, calcium, ferritin, and inflammatory markers (TNF-α, CRP, IL-1, IL-6, and IL-10) using specific analytical kits (SunLog Biotech) according to the methods described by the manufacturer.

### 2.11. Histopathological Examinations

At 8 wpv, six female and six male animals from each vaccinated group were euthanized using CO_2_ for histopathological examinations. The immune organs, i.e., lymph nodes (both local and distant from the site of administration), thymus, spleen, bone marrow, and Peyer’s patches or bronchus-associated lymphoid tissue, as well as pivotal organs (lungs, brain, kidneys, liver, and reproductive organs), and the site of vaccine administration were assessed. Different tissue sections from each organ were fixed in 10% formaldehyde, washed, dehydrated, and embedded in paraffin blocks. Sections of 5 µm thickness were stained with hematoxylin and eosin for histopathological examination. Ten random low microscopic fields (10×) per section were scanned for assessment of histopathological lesions. Images were captured using an image analysis system with a light microscope Olympus CX41 and SC100 video camera attached to a computer system. Photomicrographs were taken at different magnifications and processed using Adobe Photoshop version 8.0 (Adobe). A histopathological scoring for the inflammatory cellular infiltrate was used to assess severity of inflammation according to Kajon et al., 2003. Inflammation was assessed by enumerating layers of inflammatory cells and their extent around bronchioles, vessels, and alveolar wall. The score was categorizing groups into five categories (no inflammation, minimal, mild, moderate, and marked) [7].

### 2.12. Effect of Repeated Doses of Inactivated NRC-VACC-01 Vaccine in Mice

In a separate experiment, mice were allocated into three groups (*n* = 12). Mice of the first group received 300 µL IM injections of PBS. The other two groups were immunized with two doses of the inactivated NRC-VACC-01 vaccine (6 and 20 µg/300 µL, IM). A booster dose containing the same initial concentration was administered at 2 wpv. Animals were followed up for 14 weeks, and any mortality and clinical signs were recorded.

### 2.13. Ethical Approval

Animals were treated according to the national and international ethics guidelines. The study was approved by the ethics committee of NRC (Approval number: NRC-20074), and all procedures and experiments were performed according to the approved protocol.

### 2.14. Statistical Analysis

The collected data were coded, tabulated, and statistically analyzed using GraphPad Prism, version 8 (GraphPad Software). Data are presented as mean ± standard deviation (SD). Statistical analysis was performed using two-way ANOVA followed by the Tukey–Kramer multiple comparisons test to assess the difference between the various groups.

## 3. Results

### 3.1. Virus Isolation, Propagation, and Characterization

**Figure 1:**
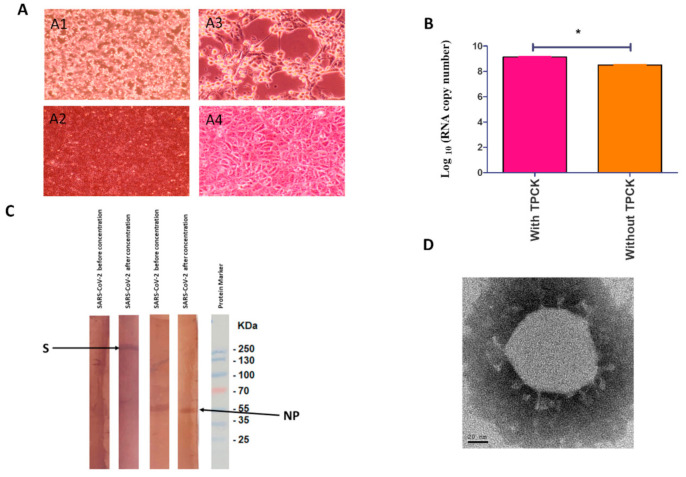
(**A**) Cytopathic effect of SARS-CoV-2 infection on African Green Monkey kidney cells (Vero-E6); (A1). Infected Vero cells on day 4 post infection with magnification 10×; (A2) infected cells at 40× magnification. A3 and A4 are Vero-E6 cells infected with the inactivated vaccine at magnification 10× and 40×, respectively. (**B**) RNA copy numbers of cultivated virus with and without TPCK-treated trypsin. The significant differences are indicated (* = *p* < 0.05). (**C**) Western blot protein profiles of cultivated viruses before and after concentration. Immunogenic peptides S and NP were characterized at about 270 and 55 kilodalton (kDa), respectively. (**D**) Electron microscope image of cultivated SARS-CoV-2 virus.

The hCoV-19/Egypt/NRC-03/2020 SARS-CoV-2 strain was successfully isolated in Vero-E6 cells from a clinical specimen. The propagated virus was further purified using plaque purification assay. The purified virus was propagated in Vero-E6 cells to generate P1 (primary vaccine seed), P2 (master vaccine seed), and P3 (virus antigen stock) in the presence and absence of TPCK-treated trypsin. CPE appeared on infected Vero-E6 cells as marked morphological changes including rounding, clumping, and darkness after 2 dpi and increased dramatically by day 4 to reach complete destruction of the cell monolayer (Figure 1A). The virus was propagated in the presence of trypsin to higher viral RNA copy number than in the absence of trypsin (*p* < 0.05) (Figure 1B). No nucleotide mutations were observed in the full genome sequences of the purified virus after propagation in Vero-E6 cells. The P3 virus was titrated by plaque titration assay. The results showed that the virus titer was 0.6 × 10^6^ PFU/mL.

### 3.2. Analysis of Viral Antigen

Staining of the concentrated and non-concentrated viral antigen by Coomassie stain showed higher intensity in the concentrated antigen. Also, WB analysis results demonstrated higher immunogenic peptides S and NP in concentrated viral antigen than non-concentrated virus (Figure 1C). Spherical viral particles with spikes in morphology with diameters of approximately 90–100 nm were shown by negative staining by electron microscopy (Figure 1D).

### 3.3. Immunogenicity of NRC-VACC-01 in Different Animal Models

#### 3.3.1. Mice and Hamsters

**Figure 2:**
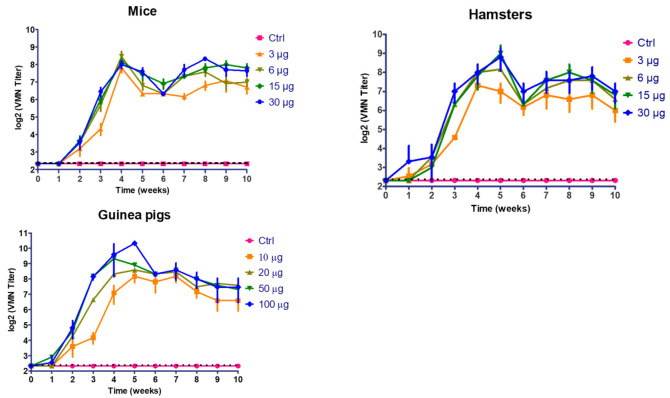
Follow up of virus microneutralization (VMN) antibody titers in vaccinated mice, hamsters, and guinea pigs using different vaccine doses. (Ctrl: control).

No significant differences (*p* > 0.05) were observed among all groups at the first and second wpv. At 3 to 10 wpv, vaccinated mice and hamsters showed significant immunological response compared with the non-vaccinated group (*p* < 0.05). Slight drops in VMN titer were observed in all vaccinated groups of mice at 5 and 6 wpv followed by an increase at 8 wpv (Figure 2). Vaccinated hamsters with 6, 15, and 30 µg of inactivated vaccines showed a peak of VMN titer at 5 wpv that slightly dropped about 2 folds during 6 to 10 wpv. Vaccinated hamsters with the lowest dose of inactivated vaccine (3 µg) developed a peak of recorded titer at 4 wpv that slightly dropped during further weeks. A significant difference (*p* < 0.05) was observed between vaccinated hamster with high (30 µg) and low dose (3 µg) at 3, 5, 7, and 8 wpv.

#### 3.3.2. Guinea Pigs

No significant differences (*p* > 0.05) were observed among all groups at the first and second wpv. At 3 to 10 wpv, vaccinated animals showed significant immunological response compared with non-vaccinated animals in a dose-dependent fashion. At week 3 to 6 post vaccination, vaccinated guinea pigs with high dose (100 µg) developed higher significant viral neutralization titer than vaccinated animals’ low dose (10 µg) (*p* < 0.05). At 7 to 10 wpv, vaccinated animals showed significant immunological response compared with control group without any significant difference among the 4 vaccinated groups (*p* > 0.05) (Figure 2).

#### 3.3.3. Rats

**Figure 3:**
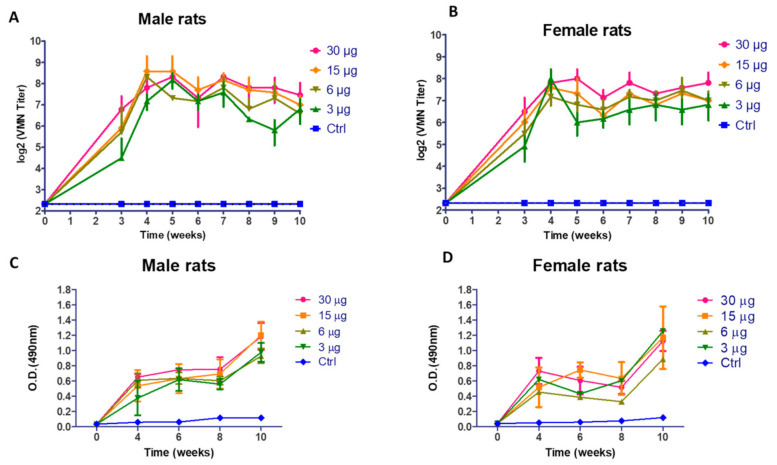
(**A**) Virus microneutralization antibody (VMN) titers in vaccinated male rats; (**B**) Virus microneutralization antibody titers in vaccinated female rats; (**C**) Optical density reading obtained for ELISA of SARS-CoV-2 in vaccinates male rats; (**D**) Optical density reading obtained for ELISA of SARS-CoV-2 in vaccinates female rats. (Ctrl: control).

At 3 to 10 wpv, vaccinated animals (males and females) showed a highly significant immunological response compared with the non-vaccinated group in a dose-dependent fashion. Slight fluctuation of VMN titers (*p* > 0.05) was observed in both vaccinated male and female rats (Figure 3A,B). The elicited IgG antibody response of male rats as illustrated in Figure 3C showed a highly significant (*p* < 0.001) increase in O.D reading in comparison with the control group at 4, 6, 8, and 10 wpv. Vaccinated female rats showed an incremental increase in antibody response (*p* < 0.05) at 4, 6, and 8 wpv and a highly significant increase in O.D reading at 10 wpv (*p* < 0.001), as shown in Figure 3D. At doses 15 and 30 µg, the male and female rats showed an elevated immune response in comparison with the 3 and 6 µg/dose of immunized rats.

### 3.4. Blood Analysis of Vaccinated Rats

Data were obtained from the analysis of blood samples collected on day 3 (acute post-immunization stage, indicated here as phase (I)), 2 weeks following the immunization (post-immunization short-term stage, indicated here as phase (II)), and 8 weeks following the immunization (prolonged effects phase, indicated here as phase (III)). They were compared among each phase for any significant differences between the different doses of the inactivated vaccine and, in comparison, with the normal control values.

CBC data revealed no significant differences between all the values obtained from the rats in all vaccinated groups and the normal control group in all phases. These values included RBCs count, hemoglobin concentration, hematocrit, platelet and WBCs counts, absolute lymphocytes, granulocyte counts, and their percentages (Figures S1–S4 and Tables S1–S8). Similarly, the serum biochemical analysis revealed the absence of any significant differences in the values of GOT, GPT, albumin, urea, creatinine, and calcium in all groups in the two phases. as well as serum ferritin and inflammatory markers (TNF-α, CRP, IL-1, IL-6, and IL-10) (Figures S5–S7 and Tables S9–S20).

The serum D-dimer portrayed marked elevation in rats vaccinated with 6, 12, and 30 µg in 3 days post-inoculation, an effect that was normalized later for all doses (starting from phase II for doses 6 and 12 µg, and in phase III for dose 30 µg) (Figure S8 and Table S21).

### 3.5. Clinical Assessment Findings

No mortality was observed in all groups throughout the duration of the experiment. No alteration in weekly body weight was observed in vaccinated animals compared to those of the control group during the duration of the experiment (Figure S9). In line with the body weight findings, food consumption was unaffected in all immunized rats (Figure S10). In addition, data revealed no change in the behavior of vaccinated animals compared to those of the normal control group, as well as the absence of any apparent reaction to the injection site. A slight increase in the body temperature was observed on day 2 following vaccination (Figure S11). The histopathological examination of different tissues from rats revealed no pathological manifestation in all vaccinated animals at different concentrations compared to the control group.

There were no observed chronic pathological findings detected in the lung in all groups in comparison to the control group. There were no changes in both male and female reproductive organs. The lung tissue revealed only minimal inflammatory reaction affecting the bronchial tree among the 15 and 30 μg groups (Figures S12 and S13). The lymph nodes were normal among all groups apart from the 15 and 30 μg groups, where there were scattered minimal tingible body macrophages (Figures S14 and S15). The thymic tissue showed only minimal medullary hyperplasia in the 3 μg vaccinated group with added scattered eosinophilic secretory materials in the other vaccinated groups compared to the control group (Figures S16 and S17). The spleen did not show any histopathological changes among all studied groups (Figures S18 and S19). However, chronicity of immune reaction at the site of injection was seen with evidence of phagocytic activity and fragmentation of previously noted homogenous globules (Figures S20 and S21). There were no significant histopathological changes observed in the brain, kidney, and liver tissue in all groups, whether in the vaccinated or the control groups (Figures S22–S28).

### 3.6. Effect of Repeated Doses of Inactivated NRC-VACC-01 Vaccine in Mice

**Figure 4:**
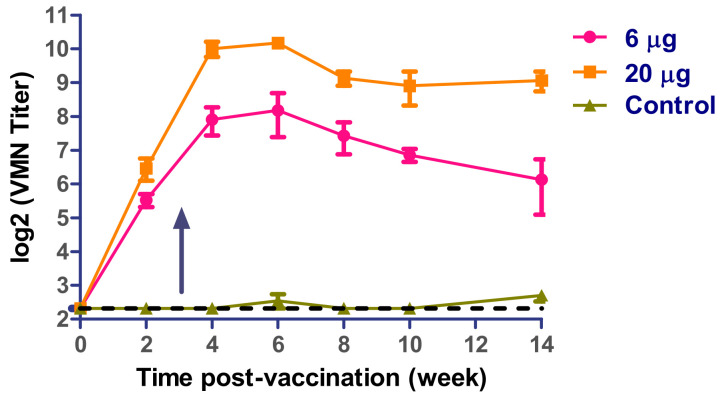
Virus microneutralization (VMN) antibody titers in prime-boost vaccinated mice. Arrow indicates booster immunization.

We measured SARS-CoV-2 neutralizing antibodies of vaccinated mice using two shots up to 14 wpv. Neutralizing antibodies to SARS-CoV-2 were first detected at week 2 after primary immunization. The VMN titer increased after a week of booster immunization to reach the peak at the 5th week. The VMN titers persisted till termination of the study (Figure 4).

### 3.7. Challenge Infection

**Figure 5:**
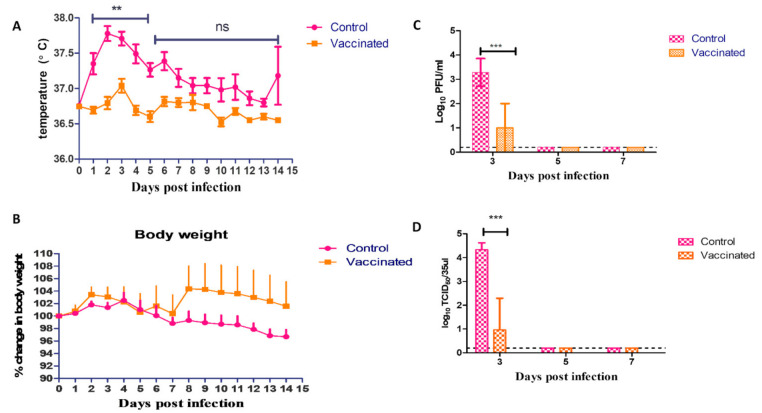
(**A**) Rectal temperature of challenged vaccinated and control hamsters; (**B**) Body weights of challenged vaccinated and control hamsters; (**C**) Viral shedding through nasal washes measured by plaque titration of challenged vaccinated and control hamsters; (**D**) Viral shedding through nasal washes measured by TCID50 of challenged vaccinated and control hamsters. The significant differences are indicated (** = *p* < 0.01, *** = *p* < 0.001 and non-significant = ns).

No mortality was observed in both groups throughout the duration of the challenge experiment. The differences between vaccinated and control groups in recorded rectal temperature were statistically significant (*p* < 0.01) at days 1 to 5 post infection, whereas, on days 6 till 14, both groups had a non-significant difference in the recorded rectal temperatures (*p* > 0.05) (Figure 5A). No significant difference in body weight was observed between the infected hamsters of the vaccinated and control groups during the fourteen days of observations. None of the vaccinated nor the control hamsters showed persistent clinical signs during all 14 days post infection (Figure 5B). On days 3, 5, and 7 after challenge, nasal washes from three hamsters of the vaccinated and control groups were collected for virus titration using plaque titration and TCID50 assay (Figure 5C,D). High virus titers were detected in the collected samples from the control group on day 3 after the challenge test as compared to the vaccinated group (*p* < 0.001). The virus was not detected in nasal samples collected on days 5 and 7 either in the control or vaccinated groups. The virus exhibited differing degrees of replication in hamster organs of the control group. High virus titers were detected in the lungs of the control group on day 3 after the challenge, as compared to the vaccinated group (*p* < 0.001) using both titration methods. The virus was not detected in both groups on days 5 and 7 in the lung. The virus was detected in the heart, intestine, and brain with limited viral titers in only infected hamsters of the control group (Figures S29 and S30). A virus neutralization assay was used to assess serum samples for seroconversion of control and vaccinated hamsters post challenge infection on day 14. Infected hamsters in the unvaccinated control group showed seroconversion after 2 weeks of infection (VMN titer > 320), while the vaccinated hamsters showed VMN titer > 640.

**Figure 6:**
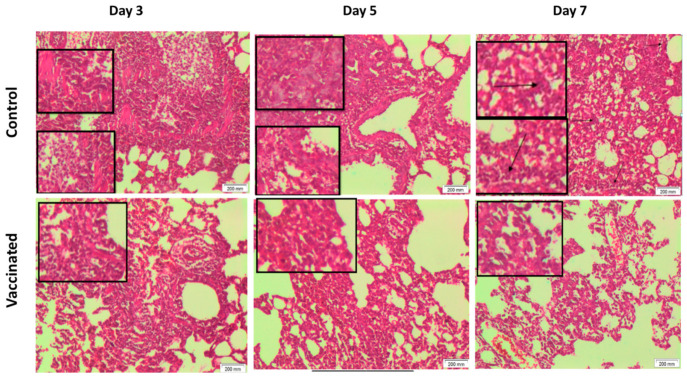
Histopathological examination of lungs of vaccinated and control challenged hamsters at different time points (H&E 100×). Vaccinated group showed better toleration and less inflammation after challenge viral infection than control group. Control group at day 3 and day 5 showed chronic inflammatory reaction, and chronic bronchitis affecting bronchial wall dissecting muscle wall. Also, control group at day 7 showed alveolar space with moderate edema, chronic bronchitis with marked inflammatory cells. The vaccinated group at day 3 and day 5 showed chronic inflammatory reaction, alveolar space with mild edema, and chronic bronchitis. Vaccinated group on day 7 showed alveolar space with mild edema, very minimal scattered inflammatory cells.

There were no remarkable histopathological differences in all assessed organs observed between all groups before the challenge test. However, following challenge, the lungs of the control unvaccinated group revealed chronic interstitial inflammatory cells in the dissecting muscle wall in most sections, with hyperplastic alveolar lining and chronic bronchitis. These changes deteriorated with time to be more marked in the tissues collected on day 7. On the other hand, the lungs of the vaccinated animals challenged with the virus showed mild histopathological changes compared to the unvaccinated animals at the respective time points. This was in the form of decreased alveolar changes and severity of inflammation. Interestingly, these mild histopathological alterations improved on day 7 (Figure 6). On the other hand, no marked histopathological change was observed in liver and renal tissues of both vaccinated and non-vaccinated groups at the three time points, i.e., 3, 5, and 7 days post challenge.

## 4. Discussion

Vaccination is considered an important strategy for controlling viral infections, especially when there are no approved drugs to combat the emerging virus. In addition to being efficacious, safety of a vaccine is a critical factor for maintaining public trust in developed vaccines. Development of an immunogenic vaccine with a high safety profile is an urgent necessity for the control of current pandemic of SARS-CoV-2. Several approaches of SARS-CoV-2 vaccine candidates were developed based on conventional vaccine technologies (inactivated, live attenuated, subunit) as well as next generation vaccine platforms (DNA, mRNA, viral vectored based vaccines) [8]. Whole inactivated vaccine is the most common and relatively easy vaccine platform and such vaccines are usually administrated intramuscularly. It is, however, limited by the productivity of the virus in cell culture facilities as well as biosafety precautions. A beneficial feature of inactivated vaccines over other next generation vaccine platforms and subunit vaccines is that the immunogenicity is likely to target whole viral proteins including spike, envelop, and nucleoprotein.

Vero cells have been certified and utilized to produce several types of licensed vaccines against different viral diseases including poliovirus, rotavirus, and influenza vaccines [9]. Vero-E6 cells produce a high viral titer of SARS-CoV-2 compared to other mammalian cell lines [10], and this characteristic is attractive for mass production. Infection of Vero-E6 cells using SARS-CoV-2 in the presence of exogenous TPCK-treated trypsin provided higher yields. Subsequent inactivation of the propagated virus in the current study using β-Propiolactone was selected as an approved inactivating agent because it has been widely used for the inactivation of several types of viruses [11]. In line with a previous study, β-Propiolactone is shown to keep the conformational structure of SARS-CoV-2 under electron microscope without any changes in viral configuration [12].

Here, we developed and evaluated the efficacy, immunogenicity, and safety profile of an inactivated SARS-CoV-2 vaccine candidate (NRC-VACC-01), based on an Egyptian SARS-CoV-2 isolate in four mammalian species, including rats, mice, hamsters, and guinea pigs. A single immunization shot using different doses evoked neutralizing antibodies against the homologous strain of SARS-CoV-2 in different experimental animal models without immunopathological observations. However, previous preclinical studies of inactivated vaccine candidates for SARS-CoV-2 showed specific neutralizing antibodies in vaccinated animal models by two-dose immunizations [2,4]. One shot is sufficient to produce effective neutralizing antibodies for influenza viruses; however, a booster dose is highly recommended after week 3 post vaccination for maintaining high antibody titers. Data related to the immunogenicity and safety of this vaccine was comprehensively collected using a number of doses ranging from 3 µg up to 100 µg for 10 weeks of observation. From the safety point of view, the results obtained from all vaccinated animals support the safety of the tested vaccine and reveal that all doses were well tolerated without local or systemic toxic manifestations. No mortality was reported in all experimental animals throughout the whole duration of the experiments. On the other hand, a slight increase in the body temperature was observed on day 2 following vaccination in female rats. Fever is the most common, generally benign, clinical sign following immunization [13]. It occurs as result of inducing some level of inflammation that is essential for the development of adaptive vaccine immunogenicity [14]. With most vaccines, the fever begins within 12 h and lasts two to three days. However, the peak body temperature after vaccination may vary with different vaccine types. With inactivated vaccines, the fever usually occurs more rapidly after vaccination as compared to live attenuated ones [15]. The finding of our study is in line with these data.

The histopathological examination of the site of injection collected on day 3 showed inflammatory cell elements detected mainly at the higher doses of vaccine. In agreement with our findings, previous data reported that vaccines, irrespective of their composition, induce some level of inflammation at the injection site within the first hours after their administration [16]. Blood analysis showed unaffected CBC and blood chemistry at different time points post-vaccination, and for up to two months, with normal levels of inflammatory biomarkers. However, a slight elevation of D-dimer was observed in some animals vaccinated with certain doses during the first two weeks post immunization, an effect that was diminished in the following weeks. The histopathological examination of different tissues from all animals revealed no pathological manifestation in all vaccinated rodents.

D-dimer is a small protein fragment present in the blood after a blood clot is degraded by fibrinolysis [17]. It reflects an ongoing activation of the hemostatic system that is initiated by the body to limit and eventually stop any blood vessel or tissue injury by creating a blood clot at the injury site until it heals then degrades. Although it has long been accepted as a useful laboratory marker in detecting thromboembolism, D-dimer might be elevated in many non-specific conditions [18]. In the current study, the elevation of D-dimer in the early phase post-immunization might indicate the activation of the body homeostasis induced by vaccines’ inherited property as inducers of inflammatory and immunization response [14,19]. In line with our explanation, many studies showed that elevated levels of D-dimer are associated in some cases with disease activity and inflammation, rather than with the risk of venous thromboembolism [20,21]. Markedly, the normalization of its level in the later stages of the current study reflects the limitation of that effect and excludes the risk of thrombosis and/or thrombotic embolism, as the value of D-dimer eliminates the possibility of a thromboembolic event when in the normal range [18]. However, this was not observed in the case of influenza vaccine [22].

Among various small laboratory animal models that were used for testing several forms of inactivated SARS-CoV-2 vaccine in the previous studies [2,4], hamsters were used for testing SARS-CoV-2 vaccine efficacy and viral pathogenicity. However, functional studies associated with immune cell population after immunization of hamsters may be limited due to the lack of availability of reagents, particularly when compared with transgenic mice. Challenge infection was performed in hamsters and proved the high safety profile of the NRC-VACC-01 vaccine. Although no significant difference in body weight was observed between the infected hamsters of the vaccinated and control groups during the fourteen days of observations, the recorded rectal temperature were statistically significant. Additionally, the lungs of infected hamsters displayed widespread tissue damage and high viral titers, on the other hand, the immunized hamsters’ lungs showed only minor lung pathology. In accordance with our findings, previous recent studies of Syrian hamsters challenged intranasally with SARS-CoV-2 revealed an expression of mild-to-moderate disease that starts very early after infection (days 1–2 after inoculation), with signs of respiratory distress, including labored breathing. Viral RNA was readily detected in the respiratory tract and other tissues (such as the small intestine). Interestingly, after two weeks of infection, hamsters typically recovered, these data were in accordance with our current results [23,24,25,26].

Hence, we were able to develop a whole virus inactivated SARS-CoV-2 vaccine that is highly tolerable, safe, and immunogenic in preclinical trials. It is recommended that this vaccine is moved to human testing commencing with a phase I trial.

## 5. Conclusions

In conclusion, we successfully generated an inactivated SARS-CoV-2 vaccine that was shown to be safe and immunogenic in several mammalian species. This vaccine was also able to reduce morbidity in hamsters. This is a potential candidate vaccine ready for a phase I clinical trial.

## Data Availability

Data is contained within the article. Reported results can be found in supplementary materials.

## Supplementary Materials

The following are available online at https://www.mdpi.com/2076-393X/9/3/214/s1, Figure S1: RBCs and hemoglobin findings of vaccinated rats. Figure S2: hematocrit and platelets findings of vaccinated rats. Figure S3: WBCs, granulocytes percentage and granulocytes absolute findings of vaccinated rats. Figure S4: lymphocytes percentage and lymphocytes absolute findings of vaccinated rats. Figure S5: Serum GOT, GPT and albumin of vaccinated rats. Figure S6: Serum creatinine, urea, and calcium of vaccinated rats. Figure S7: Serum IL-1, IL-6, and IL-10 and TNF-α of vaccinated rats. Figure S8: Serum CRP, ferritin and D-dimer of vaccinated rats. Figure S9: Weekly body weight change in rats. Figure S10: Food consumption in rats. Figure S11: Rectal temperature in Rats. Figure S12: Photomicrography of lung tissue (Male), for different studied groups (H&E 100×), have been sacrificed after 8 weeks from injection. Figure S13: Photomicrography of lung tissue (Female), for different studied groups (H&E 100×), have been sacrificed after 8 weeks from injection. Figure S14: Photomicrography of lymph node (Male) for different studied groups (H&E 50×,100×), have been sacrificed after 8 weeks from injection. Figure S15: Photomicrography of lymph node (Female) for different studied groups (H&E 50×,100×), have been sacrificed after 8 weeks from injection. Figure S16: Photomicrography of Thymus tissue (Male) for different studied groups (H&E 50×, 100×), have been sacrificed after 8 weeks from injection. Figure S17: Photomicrography of Thymus tissue (Female) for different studied groups (H&E 50×, 100×), have been sacrificed after 8 weeks from injection. Figure S18: Photomicrography of Spleen tissue (Male) for different studied groups (H&E 50×), have been sacrificed after 8 weeks from injection. Figure S19: Photomicrography of Spleen tissue (Female) for different studied groups (H&E 50×), have been sacrificed after 8 weeks from injection. Figure S20: Photomicrography of Muscle tissue (site of injection) (Male) for different studied groups (H&E 100×), have been sacrificed after 8 weeks from injection. Figure S21: Photomicrography of Muscle tissue (site of injection) (Female) for different studied groups (H&E 100×), have been sacrificed after 8 weeks from injection. Figure S22: Photomicrography of Kidney tissue (Male), for different studied groups (H&E 100×), have been sacrificed after 8 weeks from injection. Figure S23: Photomicrography of Kidney tissue (Female), for different studied groups (H&E 100×), have been sacrificed after 8 weeks from injection. Figure S24: Photomicrography of Liver tissue (Male), for different studied groups (H&E 100×), have been sacrificed after 8 weeks from injection. Figure S25: Photomicrography of Liver tissue (Female), for different studied groups (H&E 100×), have been sacrificed after 8 weeks from injection. Figure S26: Photomicrography of Brain, for different studied groups (H&E 100×), have been sacrificed after 8 weeks from injection. Figure S27: Photomicrography of Testes, for different studied groups (H&E 100×), have been sacrificed after 8 weeks from injection. Figure S28: Photomicrography of Ovaries, for different studied groups (H&E 100×), have been sacrificed after 8 weeks from injection. Figure S29: Viral titer of collected organs from vaccinated and control groups of infected hamsters. The viral titers were measured by plaque titration assay. Figure S30: Viral titer of collected organs from vaccinated and control groups of infected hamsters. The viral titers were measured by TCID50. Table S1: RBCs findings of vaccinated rats. Table S2: Hemoglobin findings of vaccinated rats. Table S3: Hematocrit findings of vaccinated rats. Table S4: Platelets findings of vaccinated rats. Table S5: WBCs findings of vaccinated rats. Table S6: Granulocytes % findings of vaccinated rats. Table S7: Granulocytes findings of vaccinated rats. Table S8: Lymphocytes % findings of vaccinated rats. Table S9: Serum albumin findings of vaccinated rats. Table S10: Serum calcium findings of vaccinated rats. Table S11: Serum creatinine findings of vaccinated rats. Table S12: Serum GOT findings of vaccinated rats. Table S13: Serum GPT findings of vaccinated rats. Table S14: Serum urea findings of vaccinated rats. Table S15: Serum CRP findings of vaccinated rats. Table S16: Serum ferritin findings of vaccinated rats. Table S17: Serum TNF-α findings of vaccinated rats. Table S18: Serum IL-1β findings of vaccinated rats. Table S19: Serum IL-6 findings of vaccinated rats. Table S20: Serum IL-10 findings of vaccinated rats. Table S21: Serum d-dimer findings of vaccinated rats.
