# Supplementary material for: Immunogenicity and Safety of an Inactivated SARS-CoV-2 Vaccine: Preclinical Studies
Source: Vaccines (Basel). 2021 Mar 3;9(3):214. doi: 10.3390/vaccines9030214 (PMC7999656; doi:10.3390/vaccines9030214)
Supplement: Supplementary file 1 [file vaccines-09-00214-s001.pdf]

Blood analysis of vaccinated rats

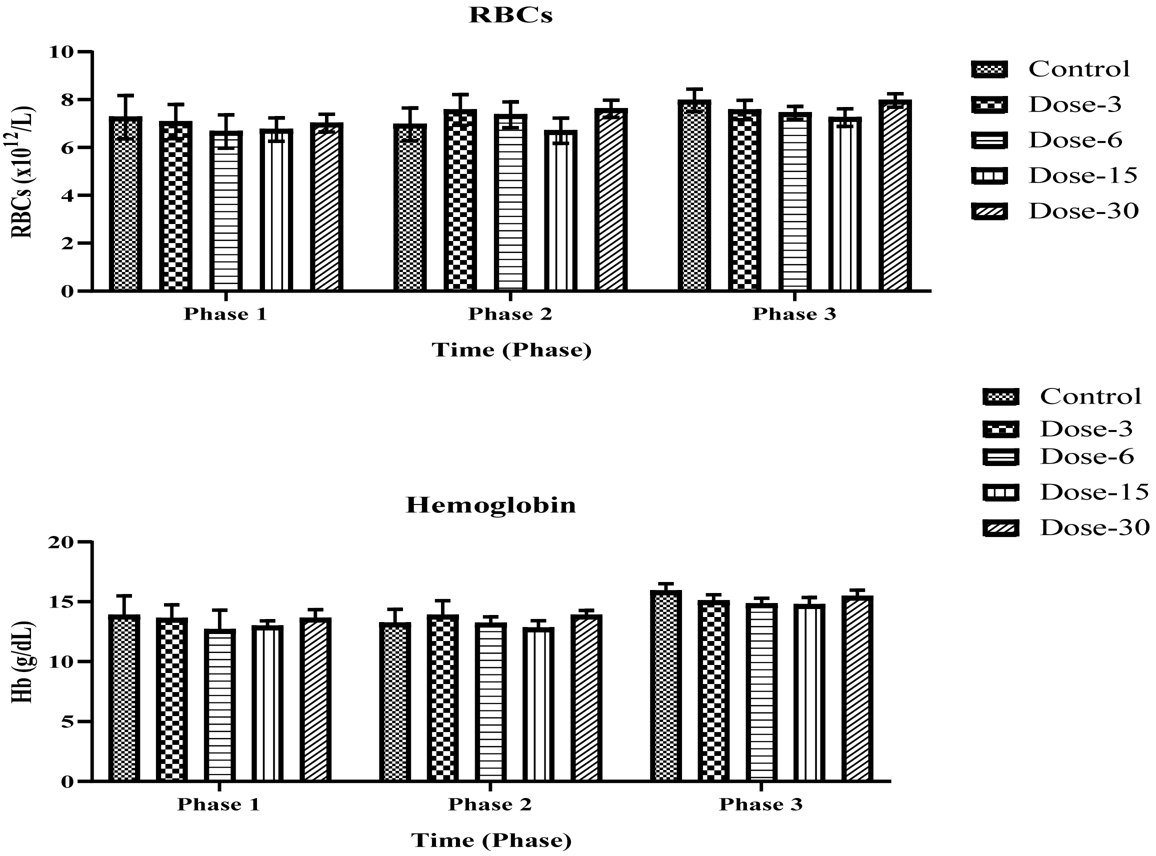

Figure S1. RBCs and hemoglobin findings of vaccinated rats
Each bar represents the mean of 5-6 rats ± SD. Statistical analysis was performed using two-way ANOVA followed by the Tukey-Kramer multiple comparisons test. (vs normal group at respective time interval) at p<0.05.*

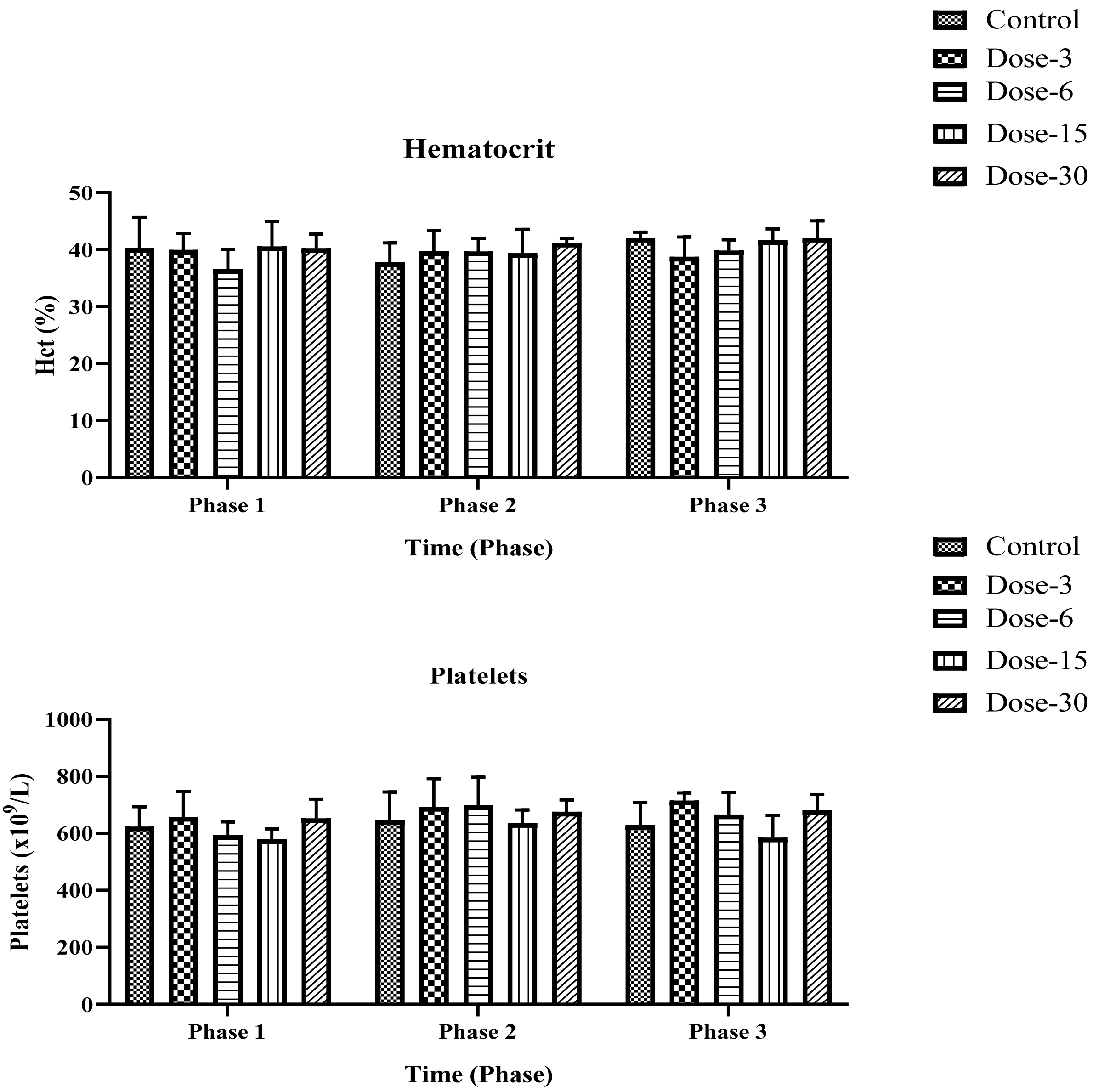

Figure S2. hematocrit and platelets findings of vaccinated rats.
Each bar represents the mean of 5-6 rats ± SD. Statistical analysis was performed using two-way ANOVA followed by the Tukey-Kramer multiple comparisons test. (vs normal group at respective time interval) at p<0.05.*

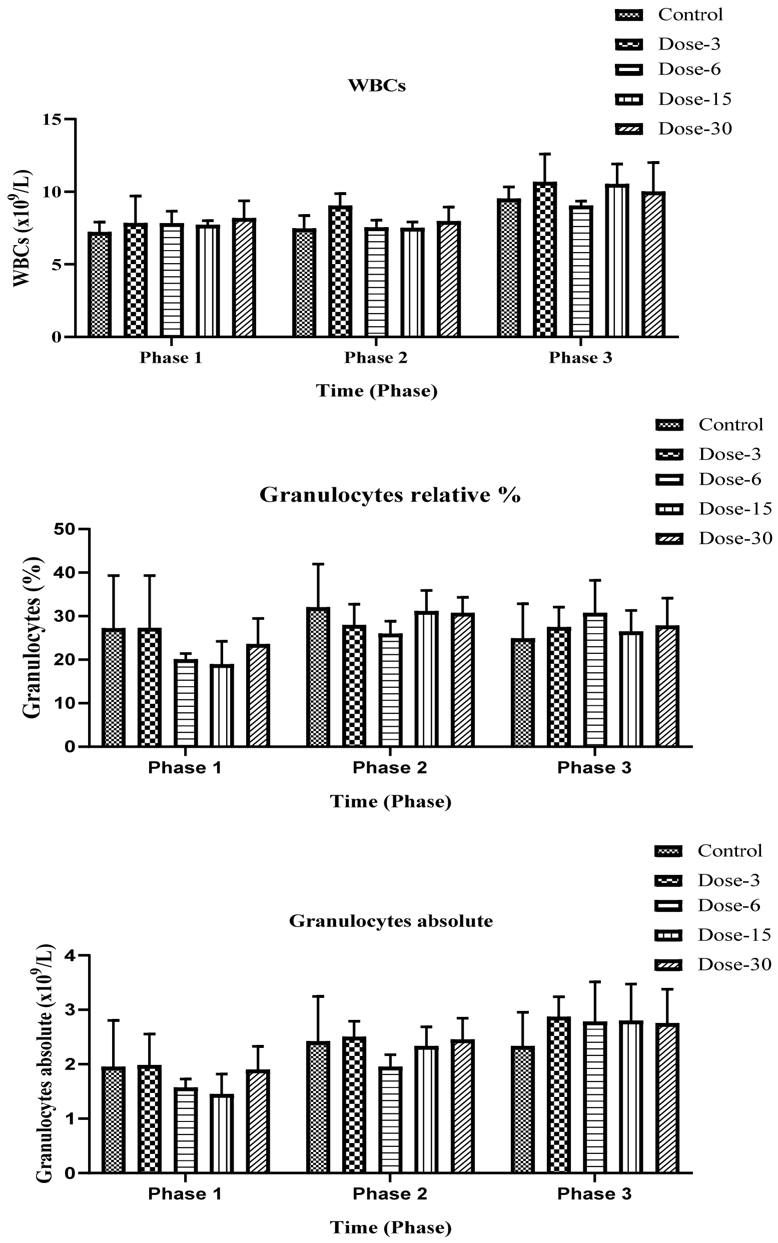

Figure S3. WBCs, granulocytes percentage and granulocytes absolute findings of vaccinated rats.

Each bar represents the mean of 5-6 rats ± SD. Statistical analysis was performed using two-way ANOVA followed by the Tukey-Kramer multiple comparisons test. (vs normal group at respective time interval) at $p<0.05$.*

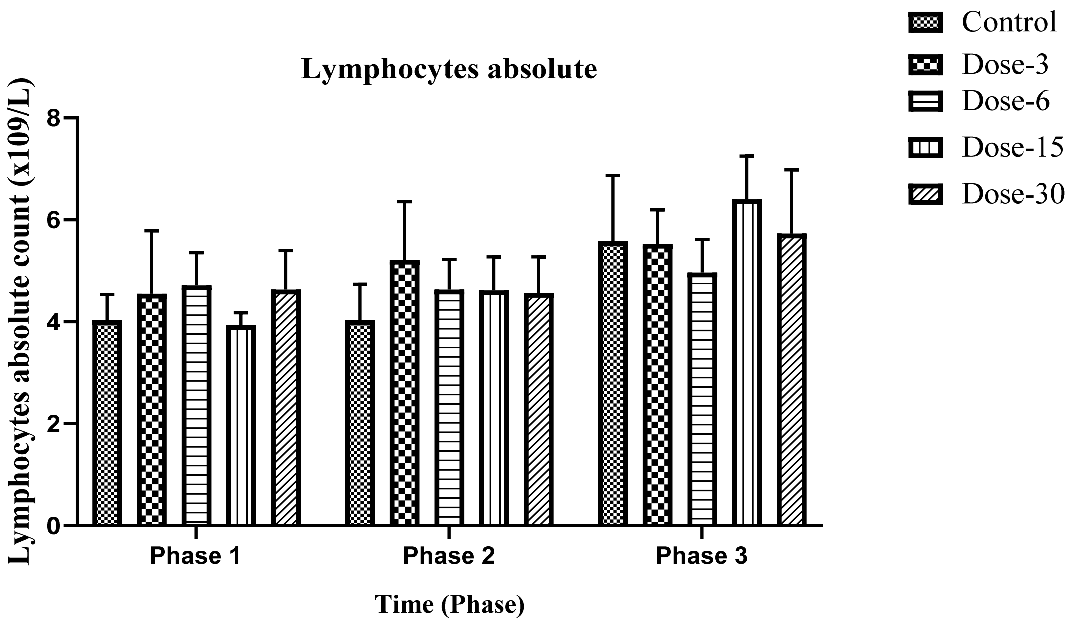

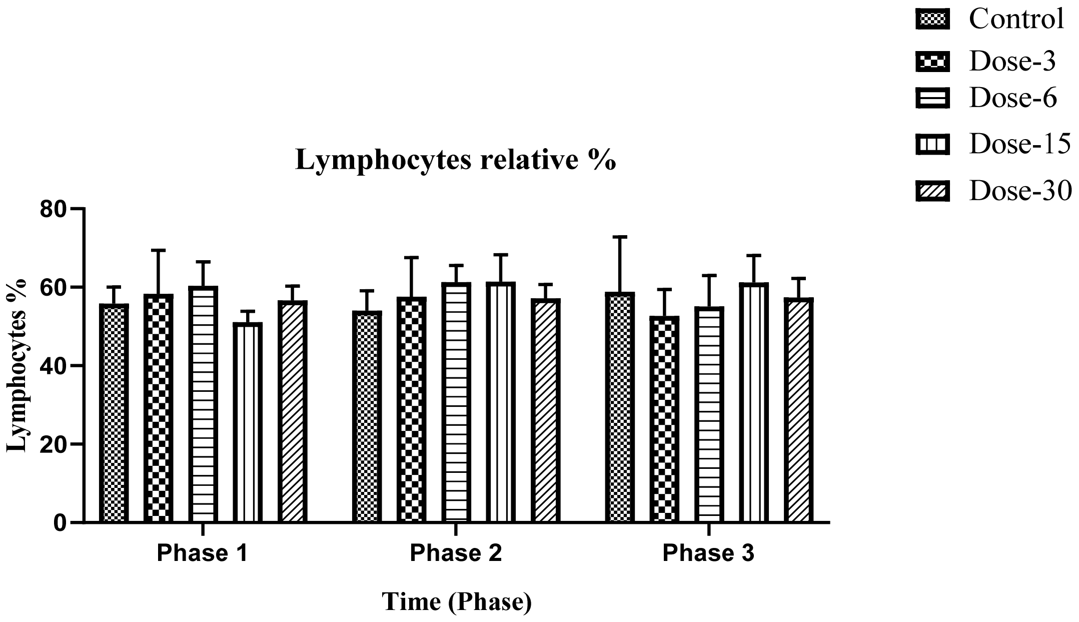

Figure S4. lymphocytes percentage and lymphocytes absolute findings of vaccinated rats.
Each bar represents the mean of 5-6 rats ± SD. Statistical analysis was performed using two-way ANOVA followed by the Tukey-Kramer multiple comparisons test. (vs normal group at respective time interval) at p<0.05.*

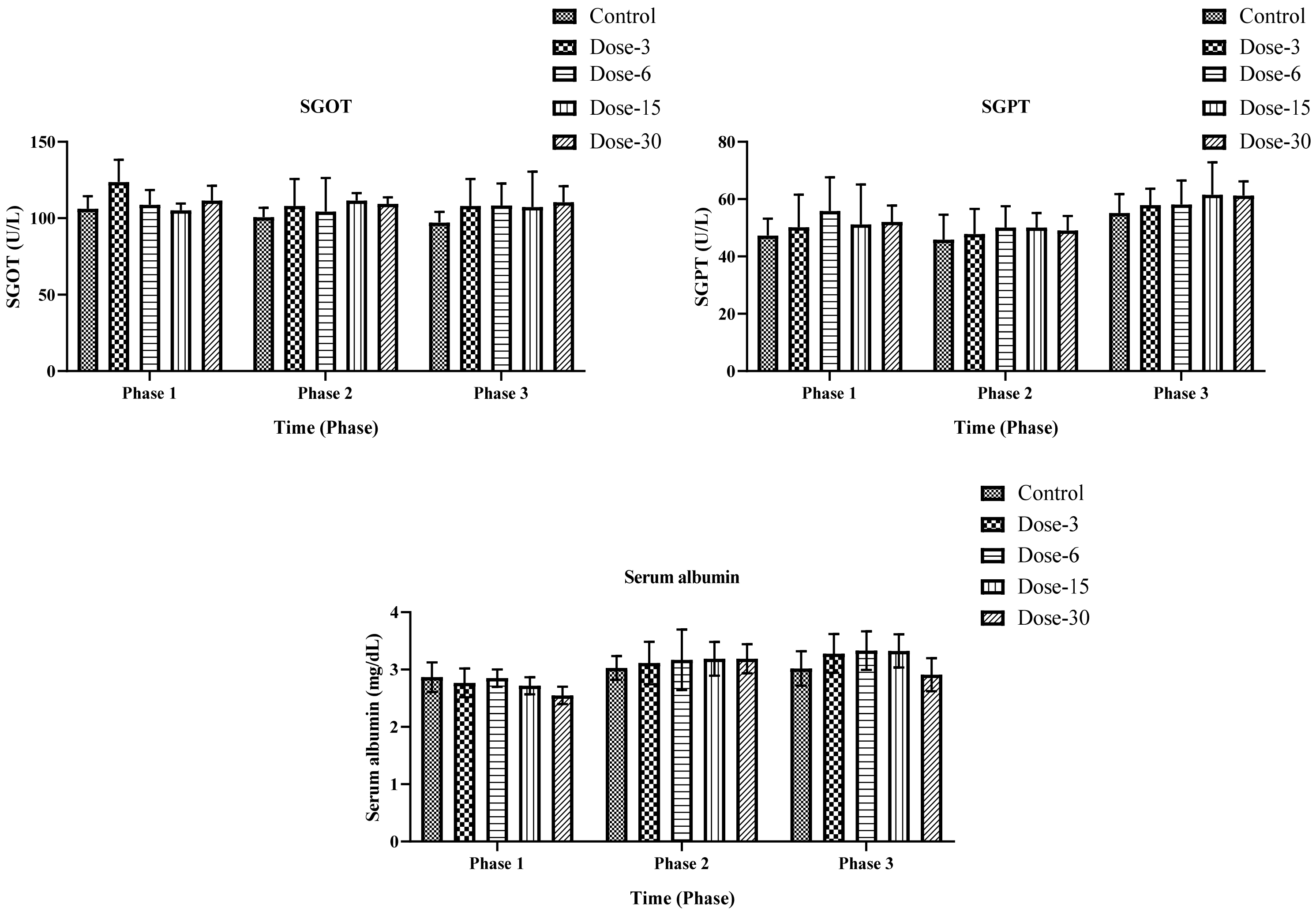

Figure S5. Serum GOT, GPT and albumin of vaccinated rats.

Each bar represents the mean of 5-6 rats ± SD. Statistical analysis was performed using two-way ANOVA followed by the Tukey-Kramer multiple comparisons test. (vs normal group at respective time interval) at p<0.05.*

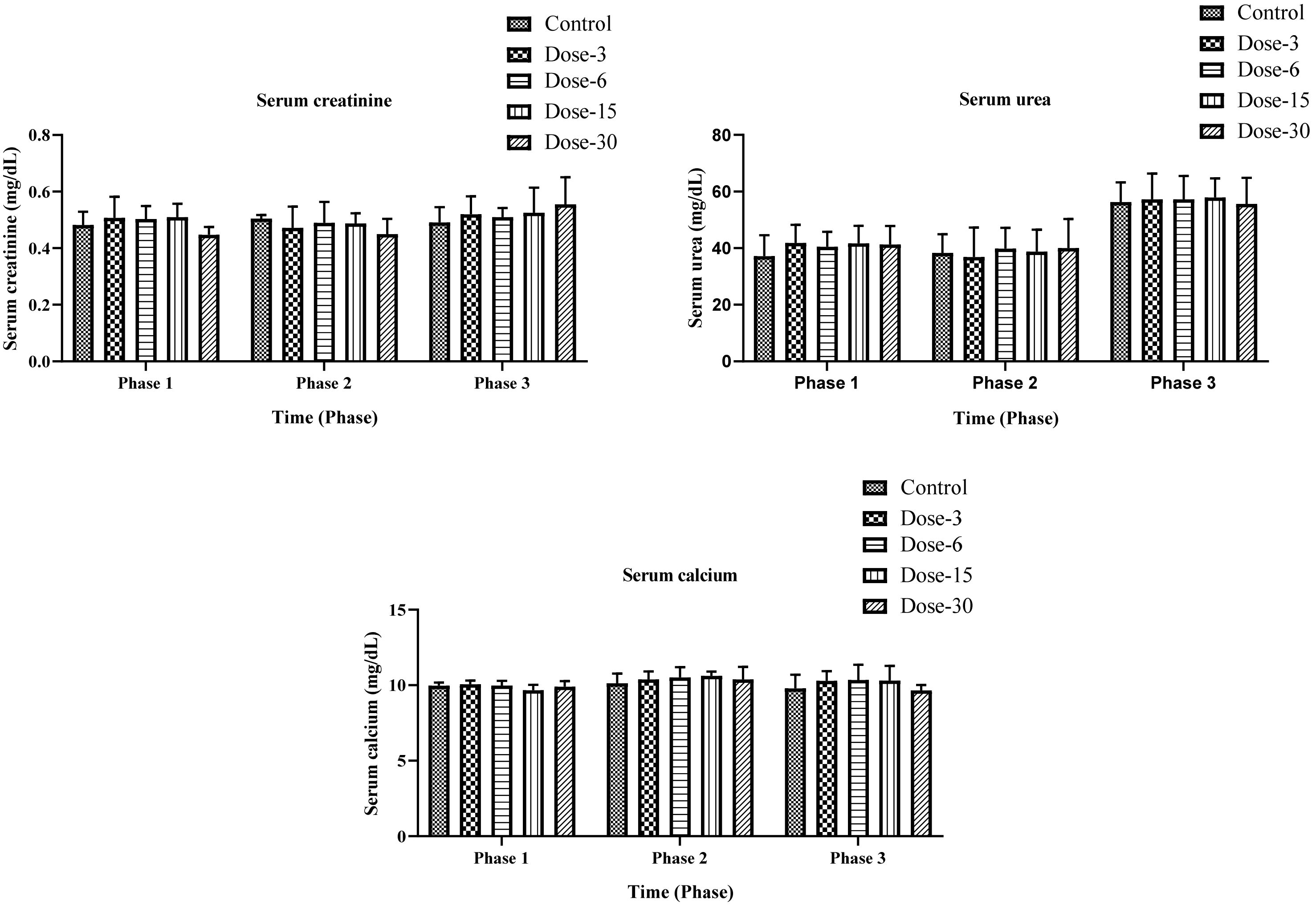

Figure S6. Serum creatinine, urea, and calcium of vaccinated rats.
Each bar represents the mean of 5-6 rats ± SD. Statistical analysis was performed using two-way ANOVA followed by the Tukey-Kramer multiple comparisons test. (vs normal group at respective time interval) at $p<0.05$.*

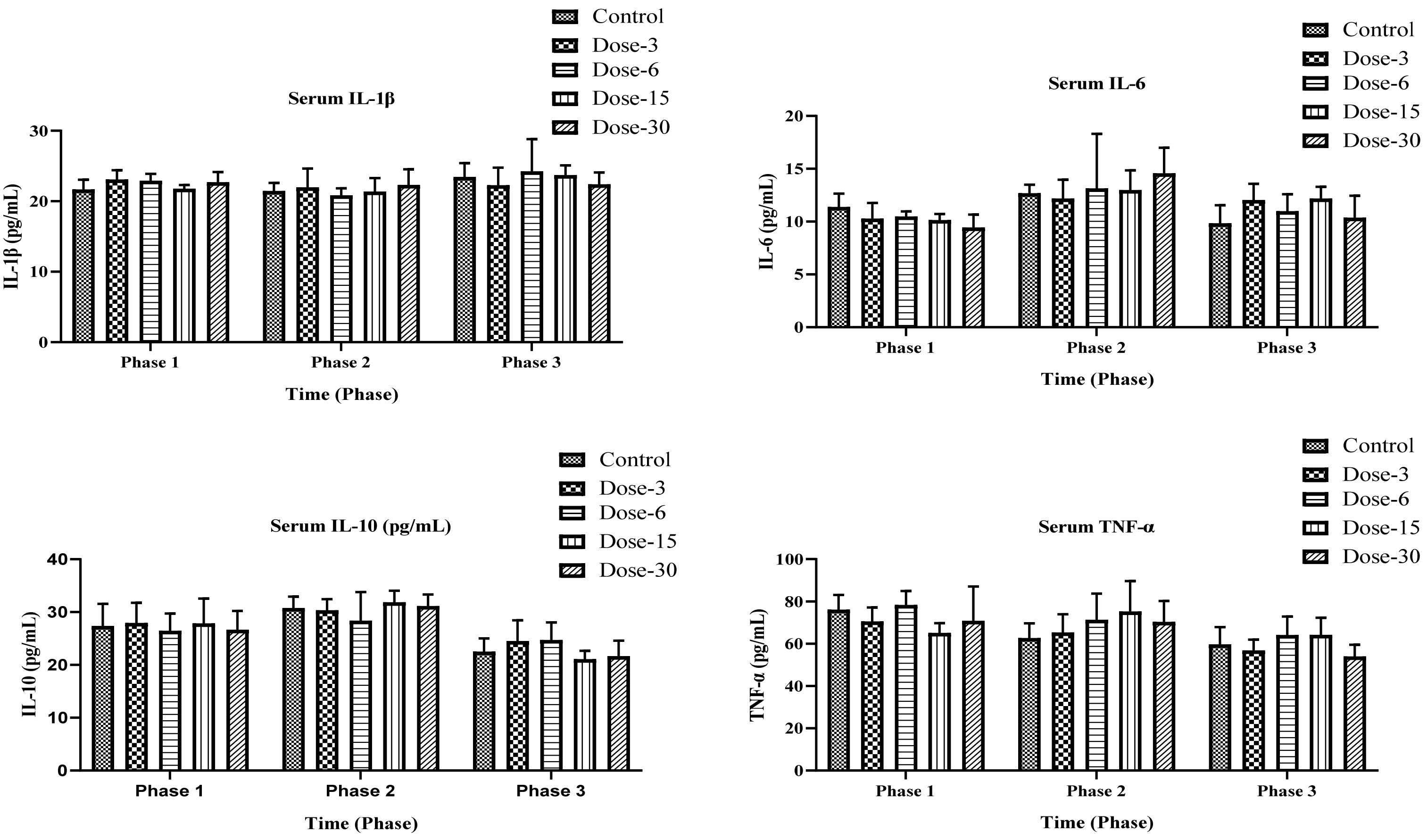

Figure S7. Serum IL-1, IL-6, and IL-10 and TNF-*α* of vaccinated rats.
Each bar represents the mean of 5-6 rats ± SD. Statistical analysis was performed using two-way ANOVA followed by the Tukey-Kramer multiple comparisons test. (vs normal group at respective time interval) at $p<0.05$.*

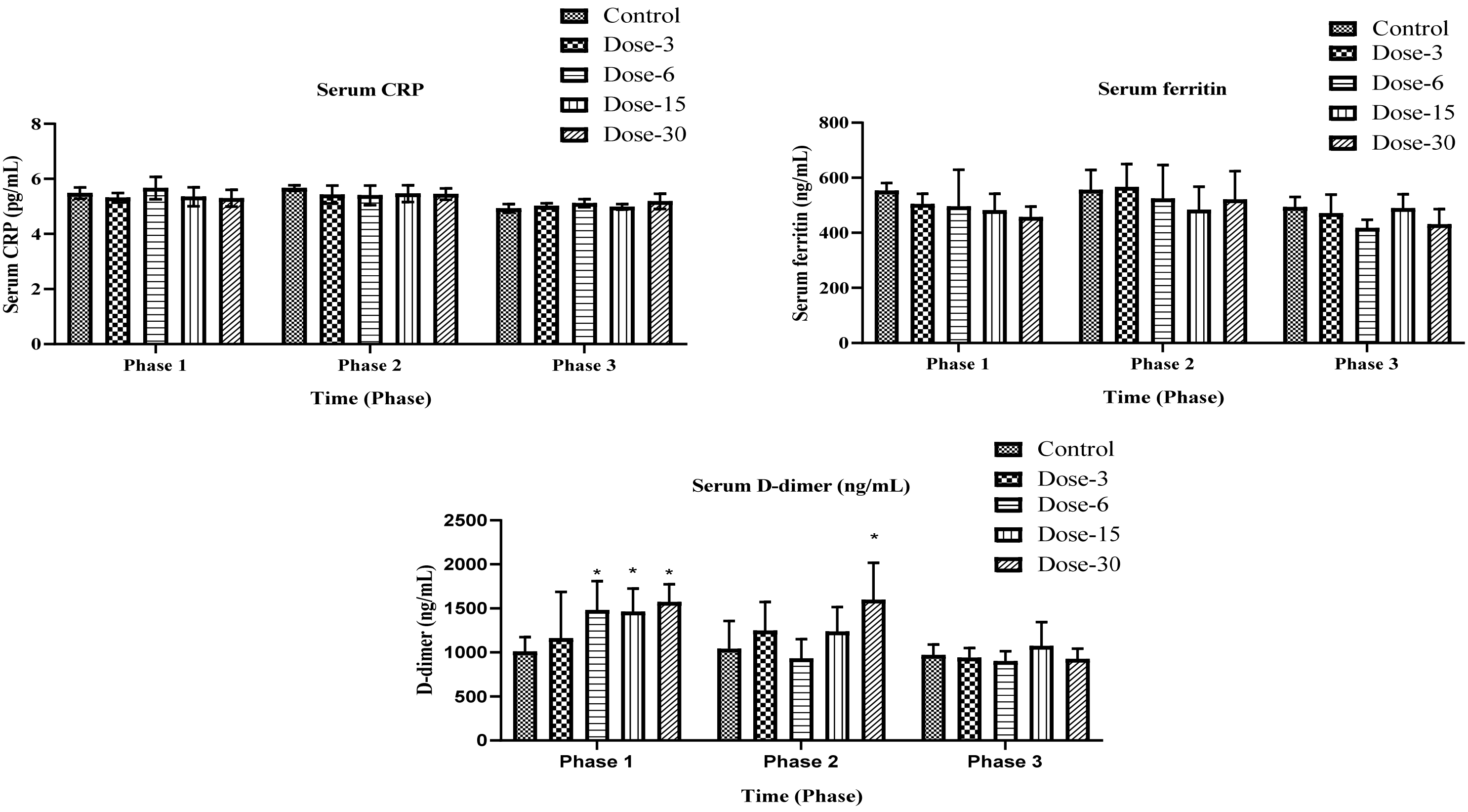

Figure S8. Serum CRP, ferritin and D-dimer of vaccinated rats.
Each bar represents the mean of 5-6 rats ± SD. Statistical analysis was performed using two-way ANOVA followed by the Tukey-Kramer multiple comparisons test. (vs normal group at respective time interval) at p<0.05.*

Clinical assessment findings

The histopathological examination of the injection site collected on day 3 showed inflammatory cell elements detected mainly at higher doses. The injection site also exhibited some foamy macrophages and foreign body pale material related to the composition and components of injected material as an immunogenic material.

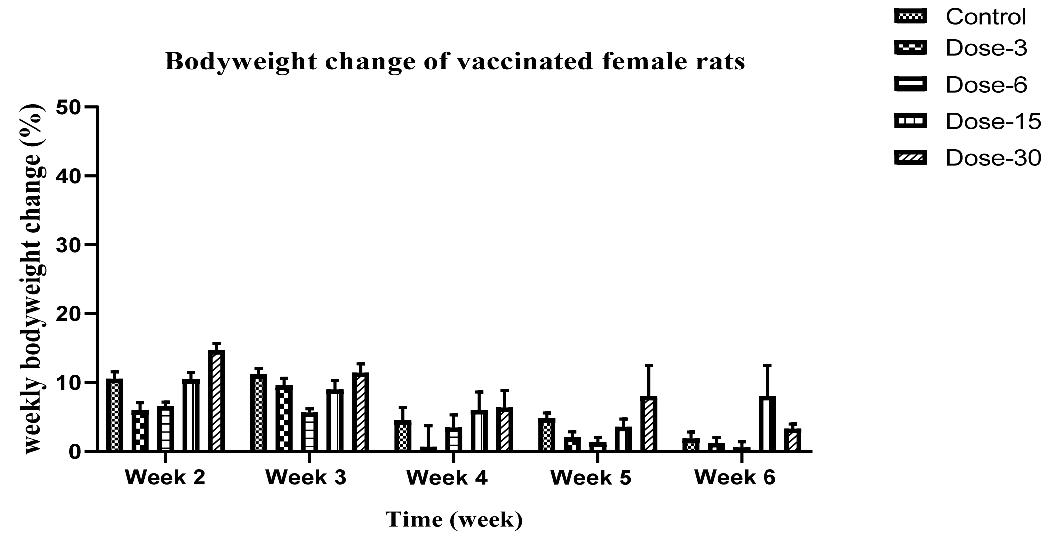

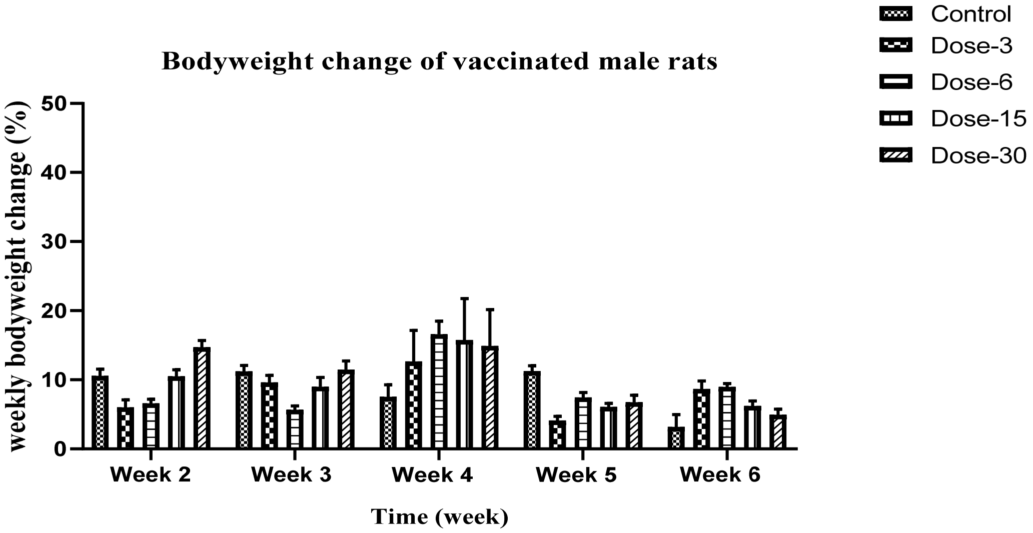

Figure S9. Weekly body weight change in r~~R~~ats.
Body weight change is calculated as a percentage from the corresponding previous week-weight. Control represents animals that received a single IM~~im~~ injection of PBS; other groups represent inoculated animals with inactivated COVID-19 vaccine in doses 3, 6, 15 and 30µL, respectively.- Each bar represents the mean of 7-11 animals ± SD. Statistical analysis was performed using two-way ANOVA followed by the Tukey-Kramer multiple comparisons test. (vs normal group at respective time interval and @ vs. day one of the same group) at $p<0.05$.*

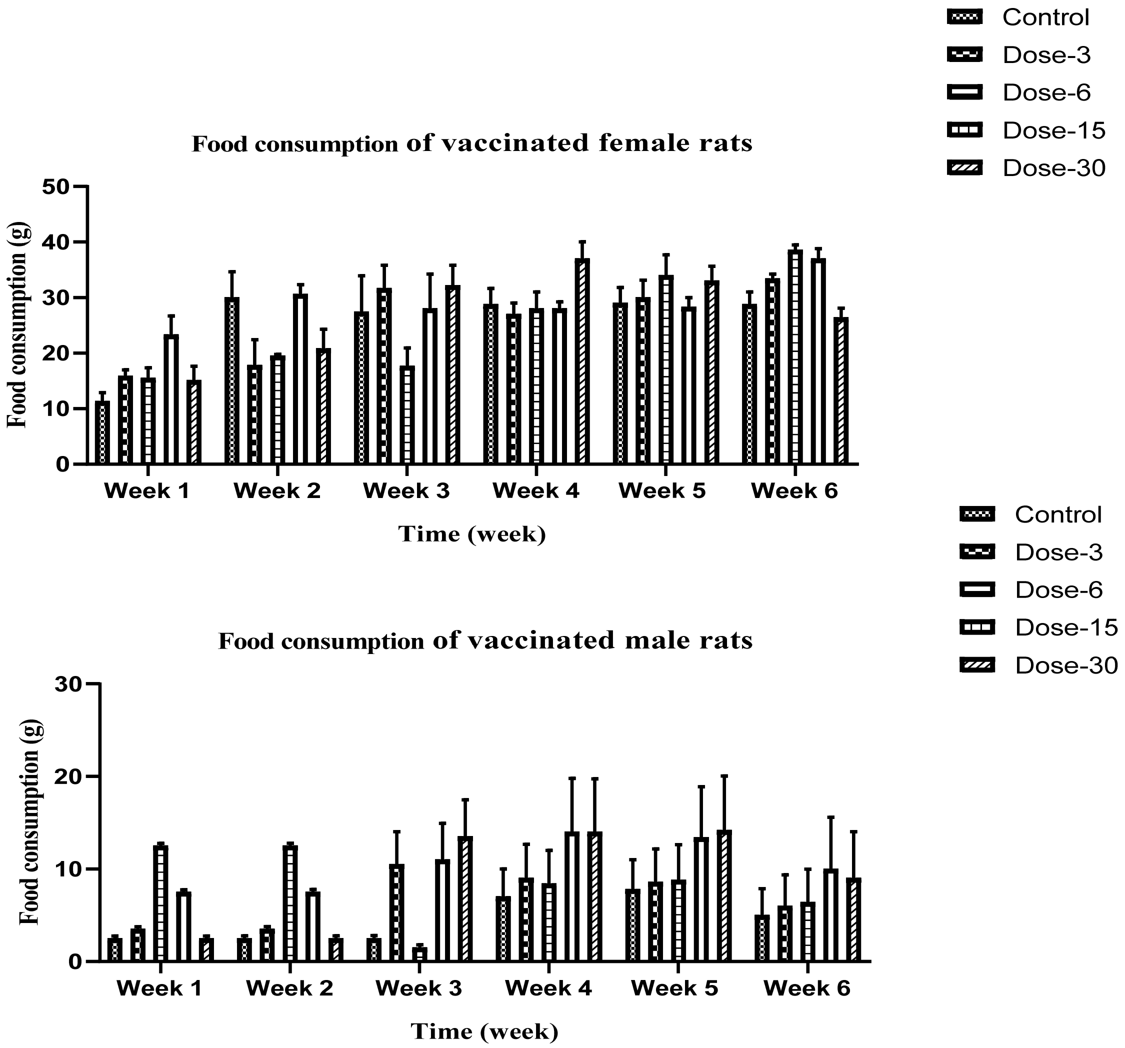

Figure S10. Food consumption in ~~R~~rats.
Control represents animals that received a single IM~~im~~ injection of PBS; other groups represent inoculated animals with inactivated COVID-19 vaccine in doses 3, 6, 15 and 30μL, respectively. ~~-~~Each bar represents the mean of 7-11 animals ± SD. Statistical analysis was performed using two-way ANOVA followed by the Tukey-Kramer multiple comparisons test. (vs normal group at respective time interval and @ vs. day one of the same group) at $p<0.05$.*

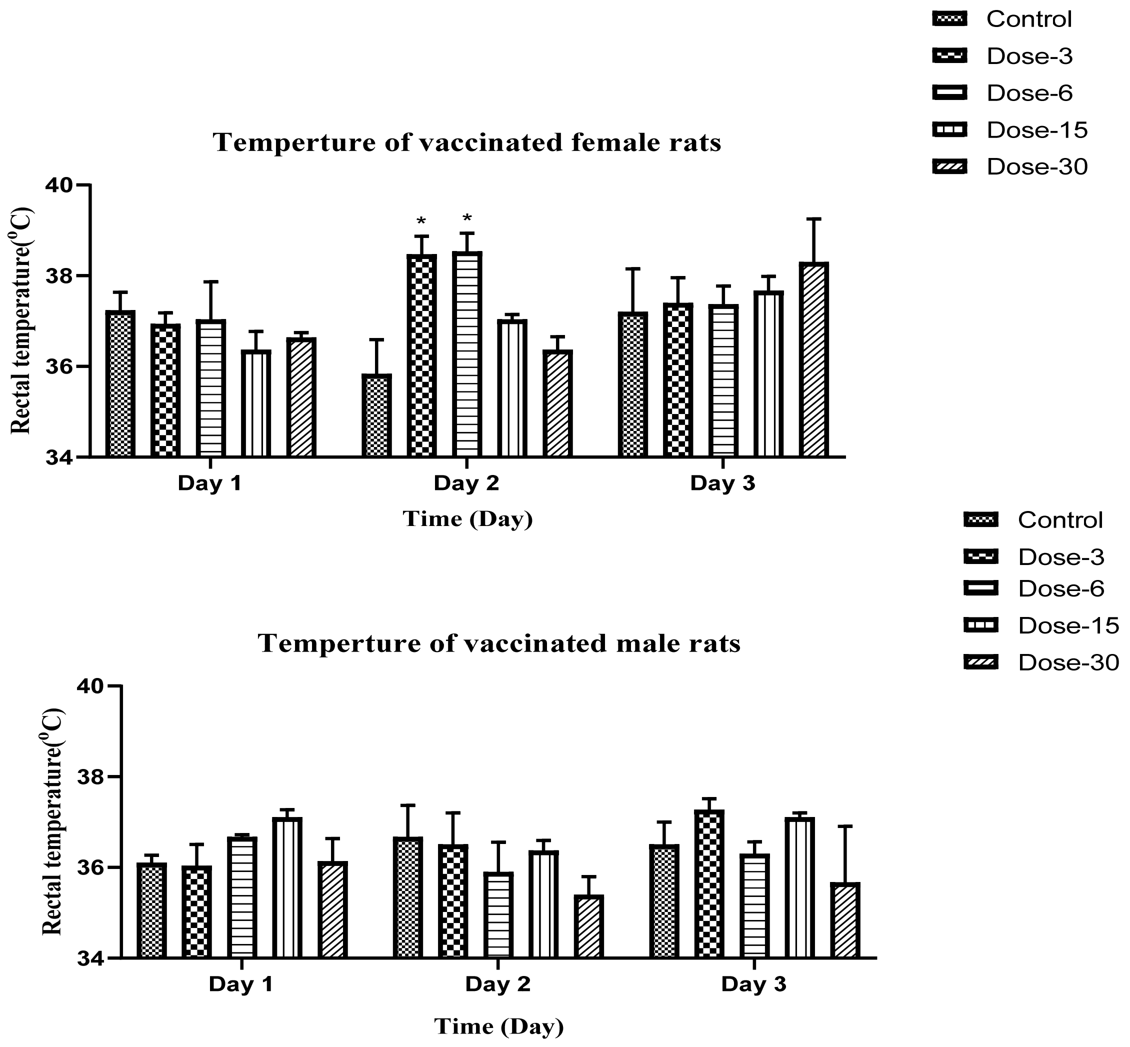

Figure S11. Rectal temperature in r~~R~~ats.
Control represents animals that received a single IM~~i.m~~ injection of PBS; other groups represent inoculated animals with inactivated COVID-19 vaccine in doses 3, 6, 15 and 30µL, respectively.- Each bar represents the mean of 7-11 animals ± SD. Statistical analysis was performed using two-way ANOVA followed by the Tukey-Kramer multiple comparisons test. (vs normal group at the respective time interval and @ vs. day one of the same group) at $p<0.05$.*

Table S1. RBCs findings of vaccinated rats.

RBCs (x1012/L)			
**groups**	**Phase 1**	**Phase 2**	**Phase 3**
**Control**	7.27±0.90	6.97±0.69	7.97±0.47
**Dose-3**	7.08±0.71 (*P* >0.9999)	7.57±0.64 (*P* =0.8368)	7.57±0.41 (*P* =0.9937)
**Dose-6**	6.67±0.69 (*P* =0.8395)	7.37±0.54 (*P* =0.9937)	7.45±0.27 (*P* =0.9420)
**Dose-15**	6.75±0.49 (*P* =0.9420)	6.70±0.53 (*P* >0.9999)	7.25±0.37 (*P* =0.6051)
**Dose-30**	7.02±0.37 (*P*>0.9999)	7.62±0.36 (*P* =0.7487)	7.98±0.28 (*P* >0.9999)

Each value represents the mean of 5-6 rats ± SD. Statistical analysis was performed using two-way ANOVA followed by the Tukey-Kramer multiple comparisons test. (vs normal group at respective time interval) at p<0.05.*

Table S2. Hemoglobin findings of vaccinated rats.

	Hb (g/dL)		
Ggroups	Phase 1	Phase 2	Phase 3
**Control**	13.87±1.62	13.23±1.15	15.90±0.60
**Dose-3**	13.60±1.15 (*P* >0.9999)	13.88±1.21 (*P* =0.9954)	15.07±0.52 (*P* =0.9573)
**Dose-6**	12.67±1.64 (*P*=0.6087)	13.20±0.55 (*P*>0.9999)	14.82±0.47 (*P* =0.7575)
**Dose-15**	12.97±0.44 (*P* =0.9236)	12.80±0.62 (*P* >0.9999)	14.75±0.62 (*P* =0.6748)
**Dose-30**	13.62±0.73 (*P* >0.9999)	13.88±0.40 (*P* =0.9954)	15.45±0.52 (*P* >0.9999)

Each value represents the mean of 5-6 rats ± SD. Statistical analysis was performed using two-way ANOVA followed by the Tukey-Kramer multiple comparisons test. (vs normal group at respective time interval) at p<0.05.*

Table S3. Hematocit findings of vaccinated rats.

	Hct (%)		
Ggroups	Phase 1	Phase 2	Phase 3
**Control**	40.28±5.36	37.77±3.43	42.07±0.98
**Dose-3**	39.95±2.92 (*P*>0.9999)	39.67±3.63 (*P* =0.9993)	38.73±3.52 (*P* =0.8899)
**Dose-6**	36.57±3.45 (*P* =0.7866)	39.63±2.37 (*P* =0.9995)	39.83±1.90 (*P* =0.9963)
**Dose-15**	40.50±4.46 (*P* >0.9999)	39.35±4.22 (*P* >0.9999)	41.65±1.98 (*P* >0.9999)
**Dose-30**	40.23±2.50 (*P* >0.9999)	41.18±0.81 (*P* =0.8707)	42.07±2.98 (*P* >0.9999)

Each value represents the mean of 5-6 rats ± SD. Statistical analysis was performed using two-way ANOVA followed by the Tukey-Kramer multiple comparisons test. (vs normal group at respective time interval) at p<0.05.*

Table S4. Platelets findings of vaccinated rats.

Platelets (x109/L)			
**Ggroups**	**Phase 1**	**Phase 2**	**Phase 3**
**Control**	623.17±70.62	644.67±100.37	628.83±80.06
**Dose-3**	657.00±89.88<br>(*P* >0.9999)	691.83±100.52<br>(*P* =0.9981)	714.50±27.36<br>(*P* =0.7581)
**Dose-6**	592.50±48.33<br>(*P* >0.9999)	698.00±99.30<br>(*P* =0.9934)	665.83±78.26<br>(*P* =0.9999)
**Dose-15**	579.00±36.80<br>(*P* =0.9991)	635.17±46.79<br>(*P* >0.9999)	584.17±79.47<br>(*P* =0.9989)
**Dose-30**	652.00±68.61<br>(*P* >0.9999)	675.00±42.07<br>(*P* >0.9999)	681.17±55.12<br>(*P* =0.9945)

Each value represents the mean of 5-6 rats ± SD. Statistical analysis was performed using two-way ANOVA followed by the Tukey-Kramer multiple comparisons test. (vs normal group at respective time interval) at p<0.05.*

Table S5. WBCs findings of vaccinated rats.

WBCs (x109/L)			
**Ggroups**	**Phase 1**	**Phase 2**	**Phase 3**
**Control**	7.22±0.69	7.45±0.90	9.52±0.82
**Dose-3**	7.83±1.87<br>(*P* =0.9998)	9.03±0.84<br>(*P* =0.5242)	10.67±1.93<br>(*P* =0.9127)
**Dose-6**	7.82±0.84<br>(*P* =0.9998)	7.53±0.51<br>(*P* >0.9999)	9.03±0.32<br>(*P* >0.9999)
**Dose-15**	7.70±0.30<br>(*P* >0.9999)	7.50±0.41<br>(*P* >0.9999)	10.52±1.39<br>(*P* =0.9701)
**Dose-30**	8.17±1.20<br>(*P* =0.9807)	7.97±0.98<br>(*P* >0.9999)	10.00±2.01<br>(*P* =>0.9999)

Each value represents the mean of 5-6 rats ± SD. Statistical analysis was performed using two-way ANOVA followed by the Tukey-Kramer multiple comparisons test. (vs normal group at respective time interval) at p<0.05.*

Table S6. Granulocytes % findings of vaccinated rats.

Granulocytes %			
**gGroups**	**Phase 1**	**Phase 2**	**Phase 3**
**Control**	27.17±12.12	32.02±9.97	24.89±7.96
**Dose-3**	27.28±12.04 (*P* >0.9999)	27.95±4.78 (*P* =0.9994)	27.45±4.61 (*P* >0.9999)
**Dose-6**	20.08±1.35 (*P* =0.9047)	25.92±2.95 (*P* =0.9694)	30.71±7.51 (*P* =0.9797)
**Dose-15**	18.95±5.29 (*P* =0.7650)	31.15±4.74 (*P* >0.9999)	26.49±4.81 (*P* >0.9999)
**Dose-30**	23.56±5.90 (*P* =0.9999)	30.73±3.56 (*P* >0.9999)	27.82±6.34 (*P* >0.9999)

Each value represents the mean of 5-6 rats ± SD. Statistical analysis was performed using two-way ANOVA followed by the Tukey-Kramer multiple comparisons test-. (vs normal group at respective time interval) at p<0.05.*

Table S7. Granulocytes findings of vaccinated rats.

Granulocytes (x109/L)			
**Ggroups**	**Phase 1**	**Phase 2**	**Phase 3**
**Control**	1.95±0.85	2.42±0.83	2.33±0.62
**Dose-3**	1.98±0.57 (*P* >0.9999)	2.50±0.29 (*P* >0.9999)	2.87±0.37 (*P* =0.9249)
**Dose-6**	1.57±0.16 (*P* =0.9957)	1.95±0.23 (*P* =0.9737)	2.78±0.73 (*P* =0.9808)
**Dose-15**	1.45±0.37 (*P* =0.9538)	2.33±0.35 (*P* >0.9999)	2.80±0.67 (*P* =0.9737)
**Dose-30**	1.90±0.43 (*P* =>0.9999)	2.45±0.39 (*P* >0.9999)	2.75±0.63 (*P* =0.9904)

Each value represents the mean of 5-6 rats ± SD. Statistical analysis was performed using two-way ANOVA followed by the Tukey-Kramer multiple comparisons test-. (vs normal group at respective time interval) at p<0.05.*

Table S8. Lymphocytes % findings of vaccinated rats.

Lymphocytes %			
Ggroups	Phase 1	Phase 2	Phase 3
Control	55.87±4.14	54.03±5.08	58.86±13.96
Dose-3	58.32±11.10 (*P* >0.9999)	57.57±9.98 (*P* >0.9999)	52.69±6.73 (*P* =0.9748)
Dose-6	60.39±6.12 (*P* =0.9987)	61.32±4.26 (*P* =0.9076)	55.10±7.87 (*P* =0.9998)
Dose-15	51.10±2.83 (*P* =0.9978)	61.43±6.88 (*P* =0.8973)	61.21±6.89 (*P* >0.9999)
Dose-30	56.65±3.68 (*P* >0.9999)	57.19±3.52 (*P* =>0.9999)	57.34±4.92 (*P* >0.9999)

Each value represents the mean of 5-6 rats ± SD. Statistical analysis was performed using two-way ANOVA followed by the Tukey-Kramer multiple comparisons test. (vs normal group at respective time interval) at p<0.05.*

Table S9. Serum albumin findings of vaccinated rats.

Serum albumin (mg/dL)			
gGroups	Phase 1	Phase 2	Phase 3
Control	2.87±0.26	3.03±0.21	3.02±0.29
Dose-3	2.77±0.25 (*P* >0.9999)	3.11±0.37 (*P* >0.9999)	3.28±0.33 (*P* =0.8272)
Dose-6	2.85±0.15 (*P* >0.9999)	3.17±0.52 (*P* =0.9998)	3.33±0.35 (*P* =0.5854)
Dose-15	2.72±0.15 (*P* >0.9999)	3.19±0.29 (*P* =0.9996)	3.32±0.29 (*P* =0.7069)
Dose-30	2.55±0.15 (*P* =0.9024)	3.18±0.25 (*P* =0.9996)	2.91±0.28 (*P* >0.9999)

Each value represents the mean of 5-6 rats ± SD. Statistical analysis was performed using two-way ANOVA followed by the Tukey-Kramer multiple comparisons test. (vs normal group at respective time interval) at p<0.05.*

Table S10. Serum calcium findings of vaccinated rats.

	Serum calcium (mg/dL)		
**Ggroups**	**Phase 1**	**Phase 2**	**Phase 3**
**Control**	9.97±0.21	10.13±0.63	9.79±0.89
**Dose-3**	10.07±0.24 (*P*>0.9999)	10.38±0.53 (*P*=>0.9999)	10.29±0.64 (*P*=0.9173)
**Dose-6**	9.98±0.31 (*P*>0.9999)	10.52±0.67 (*P*=0.9966)	10.34±1.012 (*P*=0.8437)
**Dose-15**	9.67±0.37 (*P*>0.9999)	10.61±0.29 (*P*=0.9837)	10.30±0.98 (*P*=0.9392)
**Dose-30**	9.90±0.36 (*P*>0.9999)	10.37±0.84 (*P*>0.9999)	9.65±0.37 (*P*>0.9999)

Each value represents the mean of 5-6 rats ± SD. Statistical analysis was performed using two-way ANOVA followed by the Tukey-Kramer multiple comparisons test. (vs normal group at respective time interval) at p<0.05.*

Table S11. Serum creatinine findings of vaccinated rats.

	Serum creatinine (mg/dL)		
**Ggroups**	**Phase 1**	**Phase 2**	**Phase 3**
**Control**	0.48±0.05	0.50±0.01	0.49±0.05
**Dose-3**	0.50±0.07 (*P*>0.9999)	0.47±0.07 (*P*=0.9995)	0.52±0.06 (*P*=0.9988)
**Dose-6**	0.50±0.04 (*P*>0.9999)	0.49±0.07 (*P*>0.9999)	0.51±0.03 (*P*>0.9999)
**Dose-15**	0.51±0.04 (*P*>0.9999)	0.49±0.03 (*P*>0.9999)	0.53±0.08 (*P*=0.9968)
**Dose-30**	0.44±0.02 (*P*>0.9999)	0.45±0.05 (*P*=0.9237)	0.56±0.10 (*P*=0.5218)

Each value represents the mean of 5-6 rats ± SD. Statistical analysis was performed using two-way ANOVA followed by the Tukey-Kramer multiple comparisons test. (vs normal group at respective time interval) at p<0.05.*

Table S12. Serum GOT findings of vaccinated rats.

SGOT (U/L)			
**gGroups**	**Phase 1**	**Phase 2**	**Phase 3**
**Control**	106.17±8.23	100.71±6.10	97.18±7.05
**Dose-3**	123.67±14.52 (*P*=0.6370)	108±17.64 (*P*=0.9995)	108±17.64 (*P*=0.8809)
**Dose-6**	108.83±9.64 (*P*>0.9999)	104.30±21.96 (*P*>0.9999)	108.30±14.34 (*P*=0.8579)
**Dose-15**	105.17±4.40 (*P*>0.9999)	111.50±4.93 (*P*=0.9672)	107.25±23.16 (*P*=0.9550)
**Dose-30**	111.50±9.69 (*P*>0.9999)	109.38±4.27 (*P*=0.9957)	110.40±10.59 (*P*=0.6401)

Each value represents the mean of 5-6 rats ± SD. Statistical analysis was performed using two-way ANOVA followed by the Tukey-Kramer multiple comparisons test. (vs normal group at respective time interval) at* $p<0.05$.

Table S13. Serum GPT findings of vaccinated rats.

SGPT (U/L)			
**Ggroups**	**Phase 1**	**Phase 2**	**Phase 3**
**Control**	47.33±5.85	45.85±8.72	55.18±6.60
**Dose-3**	50.17±11.39 (*P*>0.9999)	47.86±8.73 (*P*>0.9999)	57.90±5.76 (*P*>0.9999)
**Dose-6**	55.83±11.79 (*P*=0.8907)	50.10±7.42 (*P*=0.9992)	58.1±8.39 (*P*>0.9999)
**Dose-15**	51.17±13.96 (*P*=>0.9999)	50.13±5.06 (*P*=0.9995)	61.50±11.35 (*P*=0.9381)
**Dose-30**	52.00±5.76 (*P*=0.9996)	49.00±5.09 (*P*=>0.9999)	61.20±4.98 (*P*=0.9319)

Each value represents the mean of 5-6 rats ± SD. Statistical analysis was performed using two-way ANOVA followed by the Tukey-Kramer multiple comparisons test. (vs normal group at respective time interval) at $p<0.05$.*

Table S14. Serum urea findings of vaccinated rats.

Serum urea (mg/dL)			
**Ggroups**	**Phase 1**	**Phase 2**	**Phase 3**
**Control**	37.17±7.39	38.29±6.60	56.27±6.94
**Dose-3**	41.83±6.40 (*P*=0.9994)	36.86±10.46 (*P*>0.9999)	57.20±9.16 (*P*>0.9999)
**Dose-6**	40.50±5.24 (*P*>0.9999)	39.80±7.36 (*P*>0.9999)	57.20±8.27 (*P*>0.9999)
**Dose-15**	41.67±6.25 (*P*=0.9996)	38.75±7.74 (*P*>0.9999)	57.87±6.77 (*P*>0.9999)
**Dose-30**	41.33±6.47 (*P*=0.9998)	40.00±10.28 (*P*>0.9999)	55.60±9.25 (*P*>0.9999)

Each value represents the mean of 5-6 rats ± SD. Statistical analysis was performed using two-way ANOVA followed by the Tukey-Kramer multiple comparisons test. (vs normal group at respective time interval) at $p<0.05$.*

Table S15. Serum CRP findings of vaccinated rats.

Serum CRP (pg/mL)			
**gGroups**	**Phase 1**	**Phase 2**	**Phase 3**
**Control**	5.48±0.20	5.67±0.10	4.93±0.15
**Dose-3**	5.32±0.17 (*P*=0.9980)	5.43±0.32 (*P*=0.9550)	5.02±0.10 (*P*>0.9999)
**Dose-6**	5.67±0.41 (*P*=0.9947)	5.40±0.36 (*P*=0.8821)	5.12±0.15 (*P*=0.9947)
**Dose-15**	5.35±0.34 (*P*=0.9998)	5.47±0.30 (*P*=0.9880)	4.98±0.10 (*P*>0.9999)
**Dose-30**	5.30±0.30 (*P*=0.9947)	5.45±0.21 (*P*=0.9755)	5.18±0.27 (*P*=0.9243)

Each value represents the mean of 5-6 rats ± SD. Statistical analysis was performed using two-way ANOVA followed by the Tukey-Kramer multiple comparisons test. (vs normal group at respective time interval) at p<0.05.*

Table S16. Serum ferritin findings of vaccinated rats.

Serum ferritin (ng/mL)			
**Ggroups**	**Phase 1**	**Phase 2**	**Phase 3**
**Control**	652.83±28.21	355.50±73.01	193.17±36.88
**Dose-3**	604.17±37.28 (*P*=0.9980)	366.00±183.86 (*P*>0.9999)	271.17±67.93 (*P*>0.9999)
**Dose-6**	595.83±133.42 (*P*=0.9904)	324.17±122.10 (*P*>0.9999)	217.67±30.20 (*P*=0.9046)
**Dose-15**	581.67±59.75 (*P*=0.9375)	283.83±83.84 (*P*=0.9342)	189.00±51.21 (*P*>0.9999)
**Dose-30**	556.83±38.27 (*P*=0.6338)	321.33±102.30 (*P*>0.9999)	230.17±55.88 (*P*=0.9765)

Each value represents the mean of 5-6 rats ± SD. Statistical analysis was performed using two-way ANOVA followed by the Tukey-Kramer multiple comparisons test. (vs normal group at respective time interval) at p<0.05.*

Table S17. Serum TNF-α findings of vaccinated rats.

	TNF-α (pg/mL)		
Ggroups	Phase 1	Phase 2	Phase 3
Control	76.07±6.93	62.62±6.99	59.65±8.26
Dose-3	70.47±6.62 (*P*=0.9991)	65.33±8.62 (*P*>0.9999)	56.80±5.16 (*P*>0.9999)
Dose-6	78.27±6.72 (*P*>0.9999)	71.30±12.42 (*P*=0.9458)	64.07±8.77 (*P*>0.9999)
Dose-15	65.00±4.73 (*P*=0.7434)	75.20±14.41 (*P*=0.5476)	64.13±8.12 (*P*>0.9999)
Dose-30	70.82±16.21 (*P*=0.9996)	70.32±9.88 (*P*=0.9797)	53.88±5.63 (*P*=0.9988)

Each value represents the mean of 5-6 rats ± SD. Statistical analysis was performed using two-way ANOVA followed by the Tukey-Kramer multiple comparisons test. (vs normal group at respective time interval) at p<0.05.*

Table S18. Serum IL-1β findings of vaccinated rats.

	IL-1β (pg/mL)		
Ggroups	Phase 1	Phase 2	Phase 3
Control	21.67±1.39	21.43±1.18	23.43±1.97
Dose-3	23.07±1.35 (*P*=0.9966)	21.97±2.68 (*P*>0.9999)	22.27±2.49 (*P*=0.9995)
Dose-6	22.88±1.01 (*P*=0.9992)	20.82±1.02 (*P*>0.9999)	24.22±4.58 (*P*>0.9999)
Dose-15	21.75±0.55 (*P*>0.9999)	21.37±1.91 (*P*>0.9999)	23.68±1.41 (*P*>0.9999)
Dose-30	22.68±1.44 (*P*>0.9999)	22.28±2.26 (*P*>0.9999)	22.40±1.69 (*P*=0.9999)

Each value represents the mean of 5-6 rats ± SD. Statistical analysis was performed using two-way ANOVA followed by the Tukey-Kramer multiple comparisons test. (vs normal group at respective time interval) at p<0.05.*

Table S19. Serum IL-6 findings of vaccinated rats.

IL-6 (pg/mL)			
**Ggroups**	**Phase 1**	**Phase 2**	**Phase 3**
**Control**	11.37±1.27	12.68±0.81	9.82±1.72
**Dose-3**	10.28±1.48 (*P*=0.9997)	12.18±1.77 (*P*>0.9999)	12.02±1.56 (*P*=0.8388)
**Dose-6**	10.47±0.49 (*P*>0.9999)	13.13±5.18 (*P*>0.9999)	10.98±1.60 (*P*=0.9994)
**Dose-15**	10.13±0.59 (*P*=0.9989)	12.98±1.85 (*P*>0.9999)	12.18±1.11 (*P*=0.7569)
**Dose-30**	9.43±1.22 (*P*=0.9322)	14.55±2.44 (*P*=0.9479)	10.35±2.10 (*P*>0.9999)

Each value represents the mean of 5-6 rats ± SD. Statistical analysis was performed using two-way ANOVA followed by the Tukey-Kramer multiple comparisons test. (vs normal group at respective time interval) at p<0.05.*

Table S20. Serum IL-10 findings of vaccinated rats.

IL-10 (pg/mL)			
**Ggroups**	**Phase 1**	**Phase 2**	**Phase 3**
**Control**	27.35±4.20	30.75±2.16	22.48±2.54
**Dose-3**	27.92±3.82 (*P*>0.9999)	30.33±2.12 (*P*>0.9999)	24.47±3.99 (*P*=0.9994)
**Dose-6**	26.40±3.33 (*P*>0.9999)	28.35±5.43 (*P*=0.9957)	24.68±3.36 (*P*=0.9982)
**Dose-15**	27.80±4.77 (*P*>0.9999)	31.83±2.20 (*P*>0.9999)	21.05±1.64 (*P*>0.9999)
**Dose-30**	26.63±3.58 (*P*>0.9999)	31.12±2.21 (*P*>0.9999)	21.62±2.98 (*P*>0.9999)

Each value represents the mean of 5-6 rats ± SD. Statistical analysis was performed using two-way ANOVA followed by the Tukey-Kramer multiple comparisons test. (vs normal group at respective time interval) at p<0.05.*

Table S21. Serum d-dimer findings of vaccinated rats.

D-dimer (ng/ml)			
**Ggroups**	**Phase 1**	**Phase 2**	**Phase 3**
**Control**	1009.40±165.12	1040.30±317.40	970.63±119.76
**Dose-3**	1161.42±527.28 (*P*=0.8049)	1246.32±325.49 (*P*=0.7969)	940.28±110.66 (*P*=0.9919)
**Dose-6**	1495.17±230.1705* (*P*=0.0371)	929.62±221.20 (*P*=0.9739)	900.98±112.35 (*P*=0.8630)
**Dose-15**	1499.5±266.3102* (*P*=0.0351)	1235.45±280.80 (*P*=0.8264)	1071.22±272.55 (*P*=0.6517)
**Dose-30**	1572.33±202.66* (*P*=0.0136)	1596.73±420.83* (*P*=0.0423)	925.33±117.56 (*P*=0.9655)

Each value represents the mean of 5-6 rats ± SD. Statistical analysis was performed using two-way ANOVA followed by the Tukey-Kramer multiple comparisons test. (vs normal group at respective time interval) at p<0.05.*

Histopathological findings

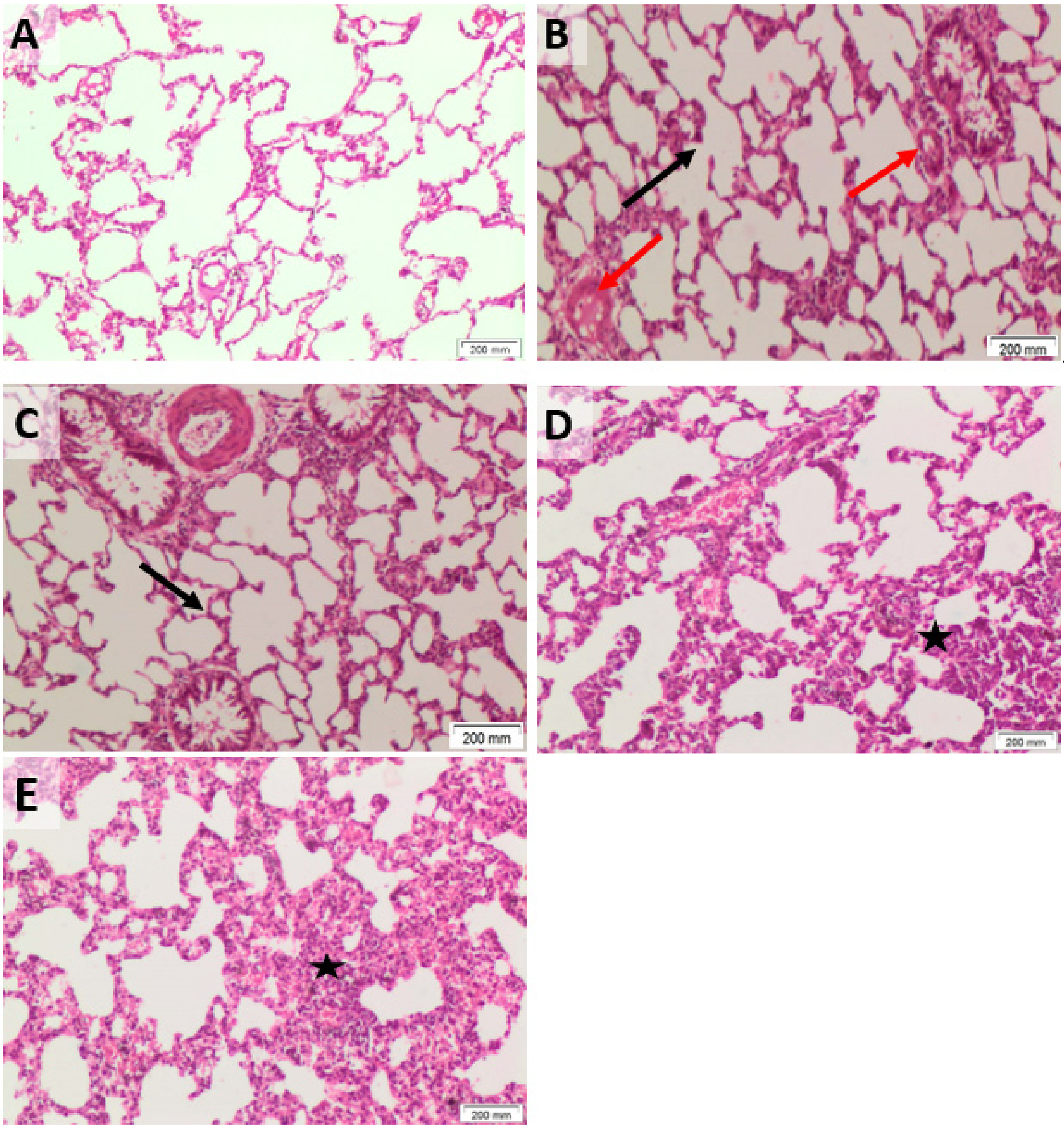

Figure S12. Photomicrography of lung tissue (Male), for different studied groups (H&E 100x), have been sacrificed after (8 weeks) from injection. A: Control group; normal appearance of alveolar walls with average thickness.
B & C: Group treated with 3 µg & 6 µg group respectively; normal appearance of alveolar walls (black arrows) average thickness of blood vessels (red arrows). D & E: Groups treated with 15 µg & 30 µg respectively: minimal inflammatory reaction (black stars) affecting bronchial wall.

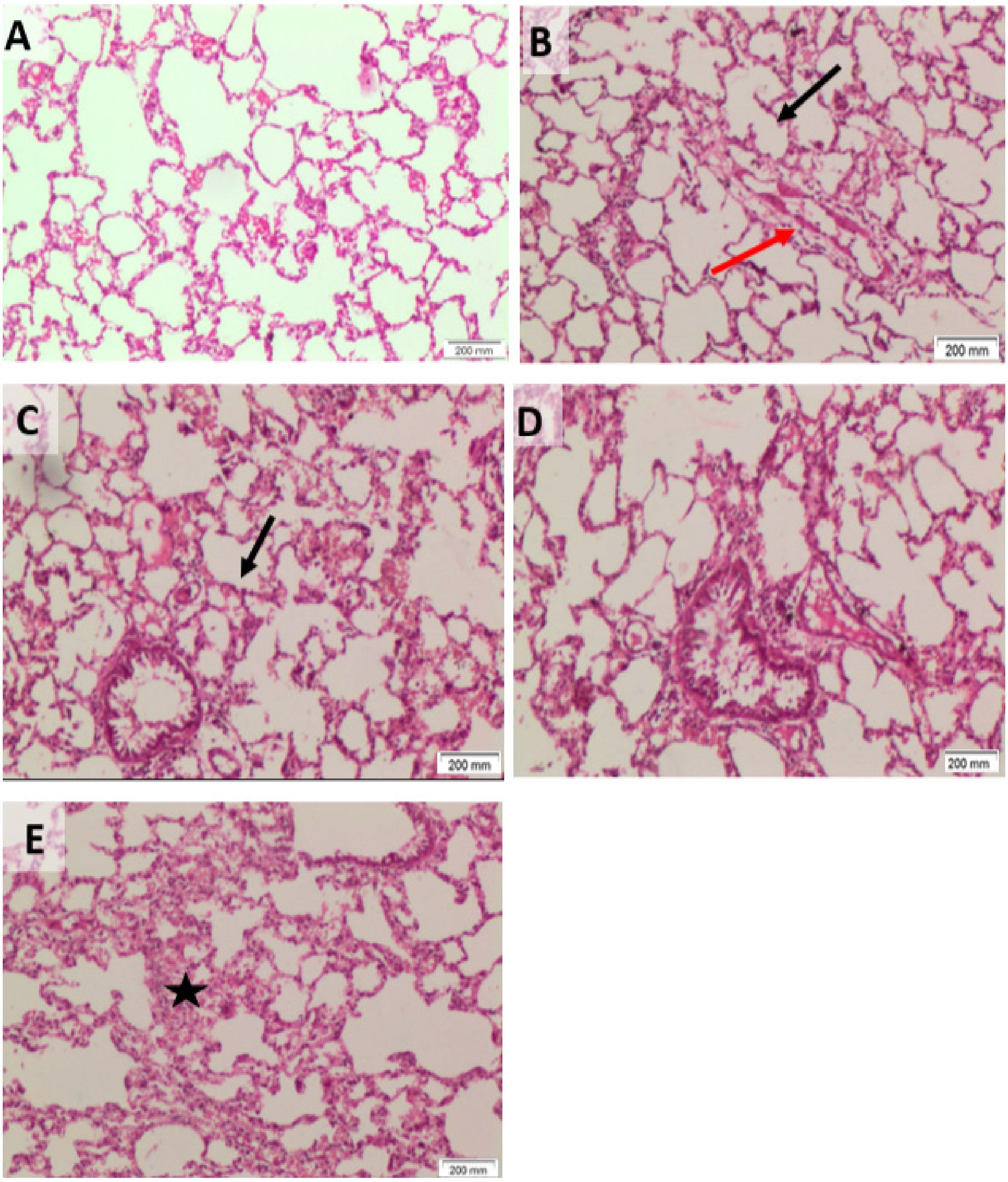

Figure S13. Photomicrography of lung tissue (Female), for different studied groups (H&E 100x), have been sacrificed after (8 weeks) from injection. A: Control group; normal appearance of alveolar walls with average thickness. B, C & D: Groups treated with 3, 6, & 15 µg group respectively; normal appearance of alveolar walls (black arrows) average thickness of blood vessels (red arrows). E;: Group treated with 30µg group: showing minimal inflammatory reaction (black stars) affecting bronchial wall.

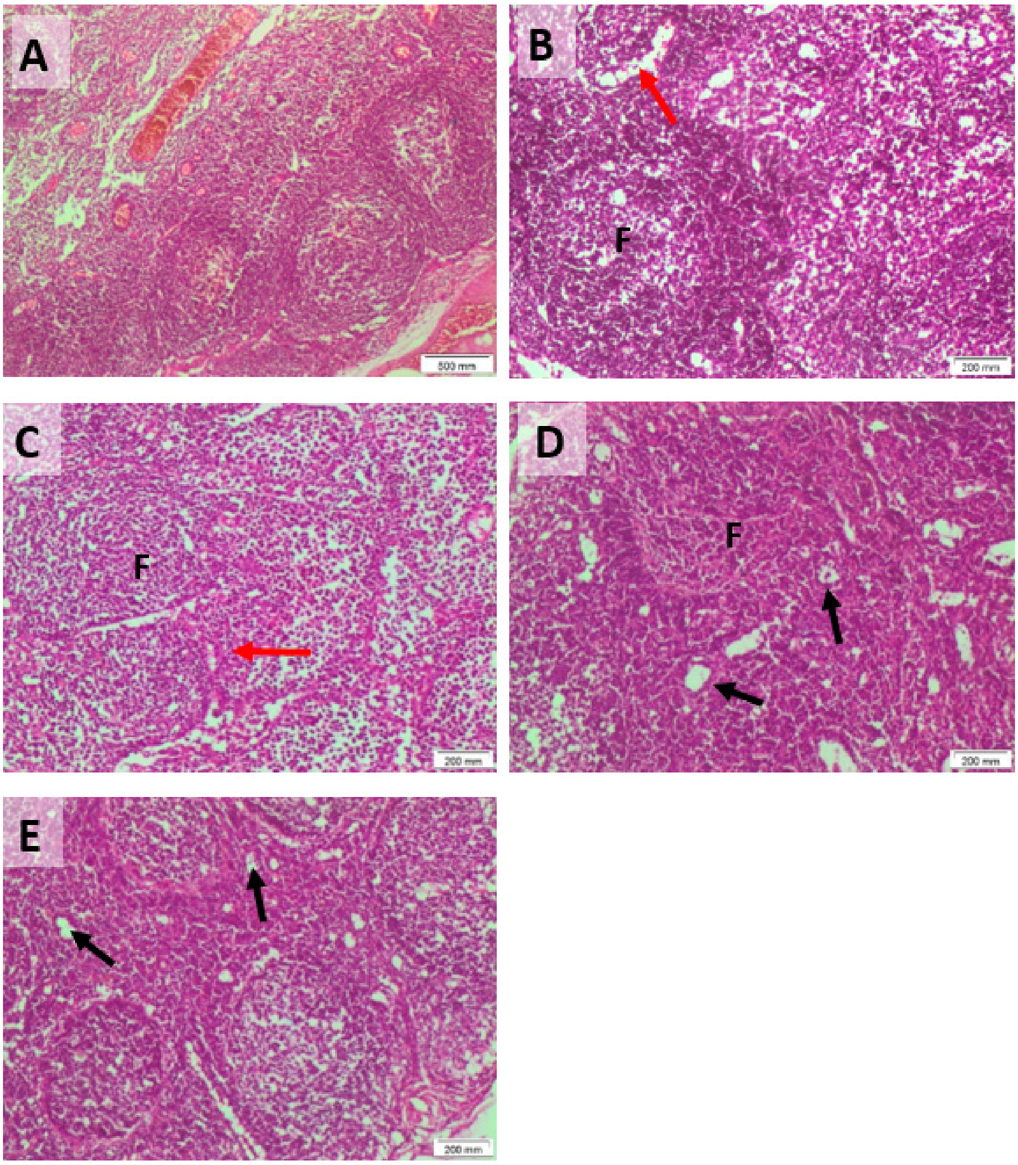

Figure S14. Photomicrography of lymph node (Male) for different studied groups (H&E 50x,100x), have been sacrificed after ~~(~~8 weeks~~)~~ from injection. A~~–~~: Control group: showing normal histological appearance. B & C: Groups~~-~~ treated with 3 & 6 µg respectively; normal histological appearance with average follicular appearance. ~~(F) with no vascular changes (red arrows).~~ D & E: Groups ~~-~~treated with 15 & 30 µg respectively; normal histological appearance with average follicular appearance (F) with no vascular changes (red arrows) scattered minimal tingable body macrophages (black arrows).

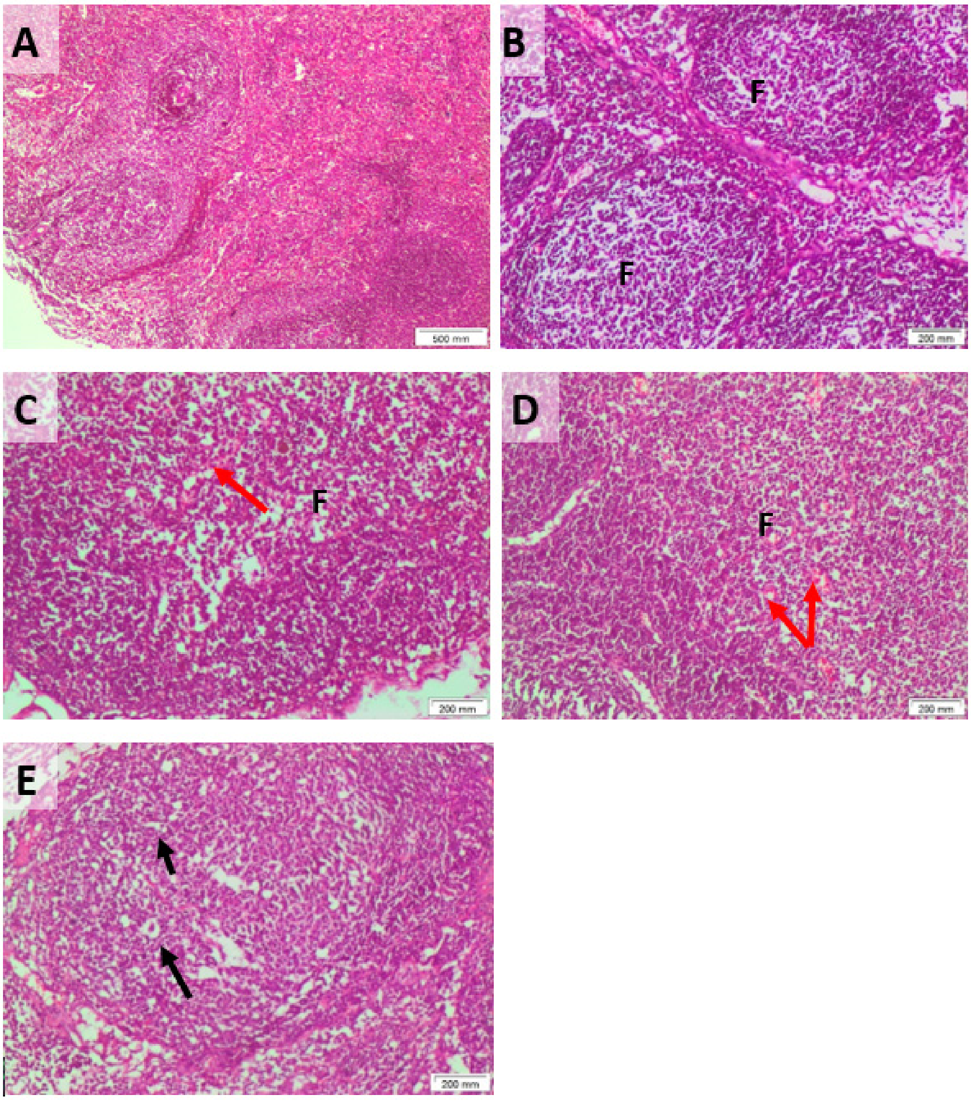

Figure S15. Photomicrography of lymph node (Female) for different studied groups (H&E 50x,100x), have been sacrificed after (8 weeks) from injection. A–: Control group: showing normal histological appearance. B & C: –Groups-treated with 3 & 6 µg respectively; normal histological appearance with average follicular appearance (F) with no vascular changes (red arrows). D & E: -Groups -treated with 15 & 30 µg respectively; normal histological appearance with average follicular appearance (F) with no vascular changes (red arrows) scattered minimal tingable body macrophages (black arrows).

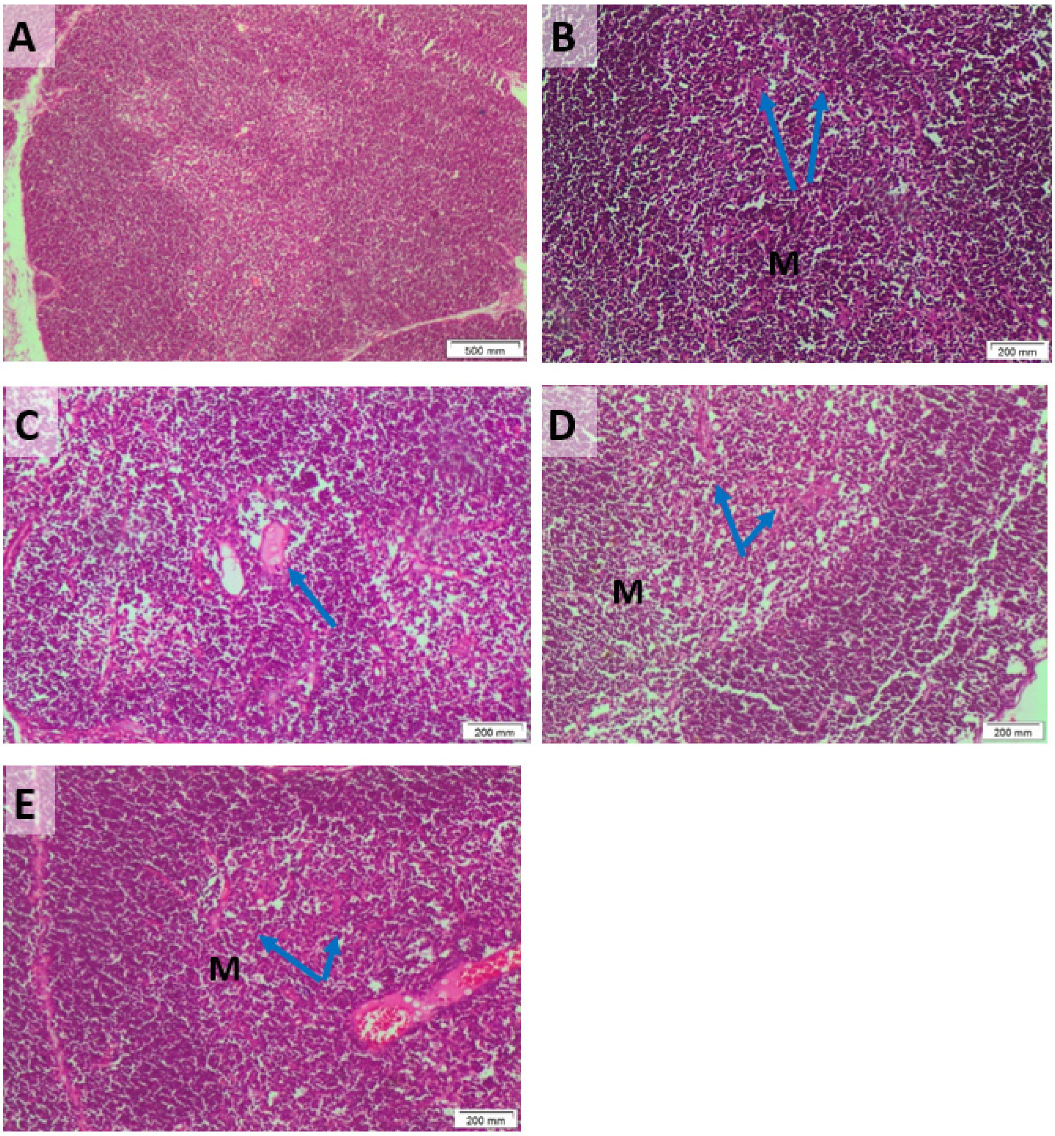

Figure S16. Photomicrography of Thymus tissue (Male) for different studied groups (H&E 50x, 100x), have been sacrificed after (8 weeks) from injection. A–: Control group: showing thymus lobules separated with fibrous strands with normal appearance of both medulla and cortex. B-: Group- treated with 3 µg: have the same picture, showing minimal medullary hyperplasia (M) and the cortex has normal appearance.C ,D & E–: Groups -treated with 6 ,15, & 30 µg respectively: showing minimal medullary hyperplasia (M) with scattered eosinophilic secretory materials (blue arrows) and the cortex has normal appearance.

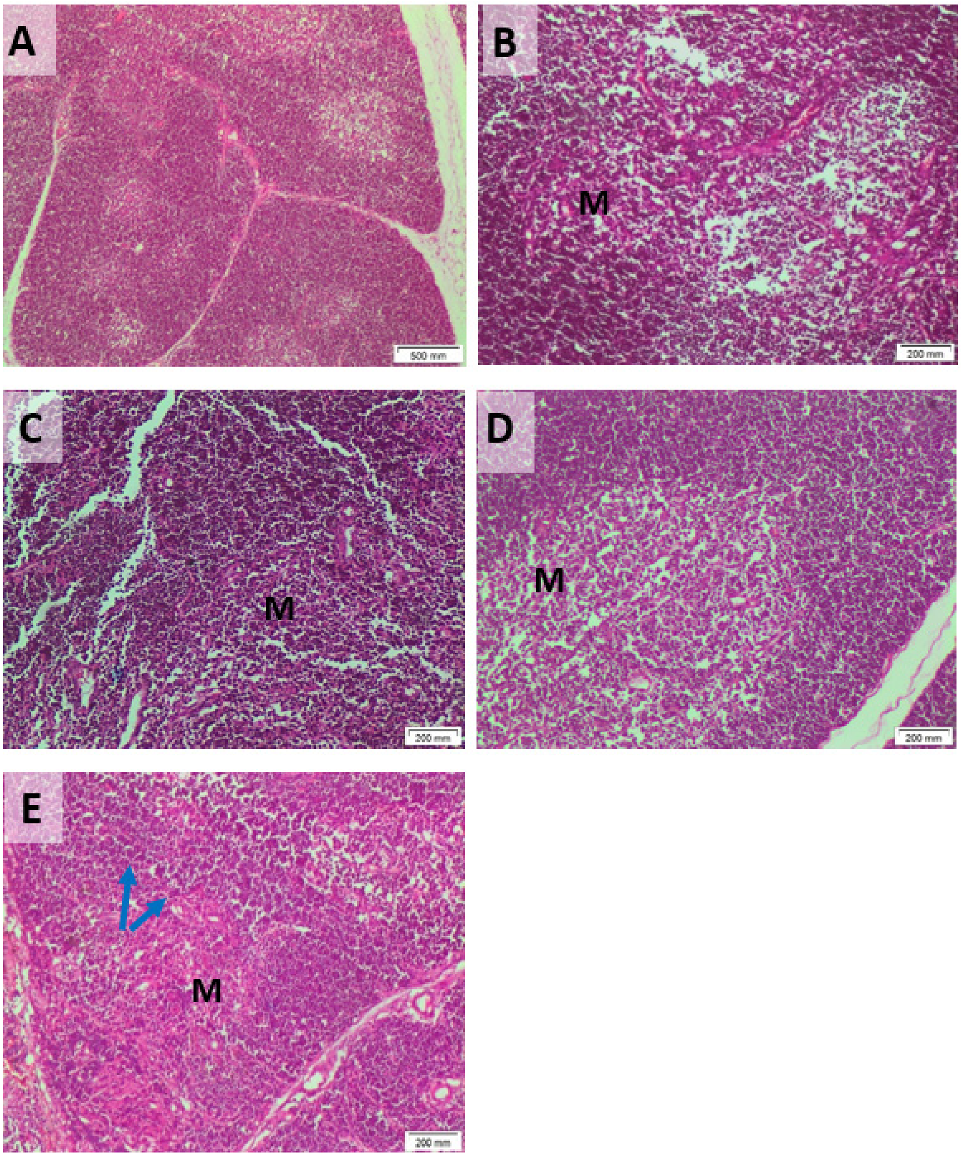

Figure S17. Photomicrography of Thymus tissue (Female) for different studied groups (H&E 50x, 100x), have been sacrificed after (8 weeks) from injection. A–: Control group: showing thymus lobules separated with fibrous strands with normal appearance of both medulla and cortex.. B–: Group -treated with 3 µg: have the same picture, showing minimal medullary hyperplasia (M) and the cortex has normal appearance. C ,D & E–: Groups -treated with 6 ,15, & 30 µg respectively: showing minimal medullary hyperplasia (M) with scattered eosinophilic secretory materials (blue arrows) and the cortex has normal appearance.

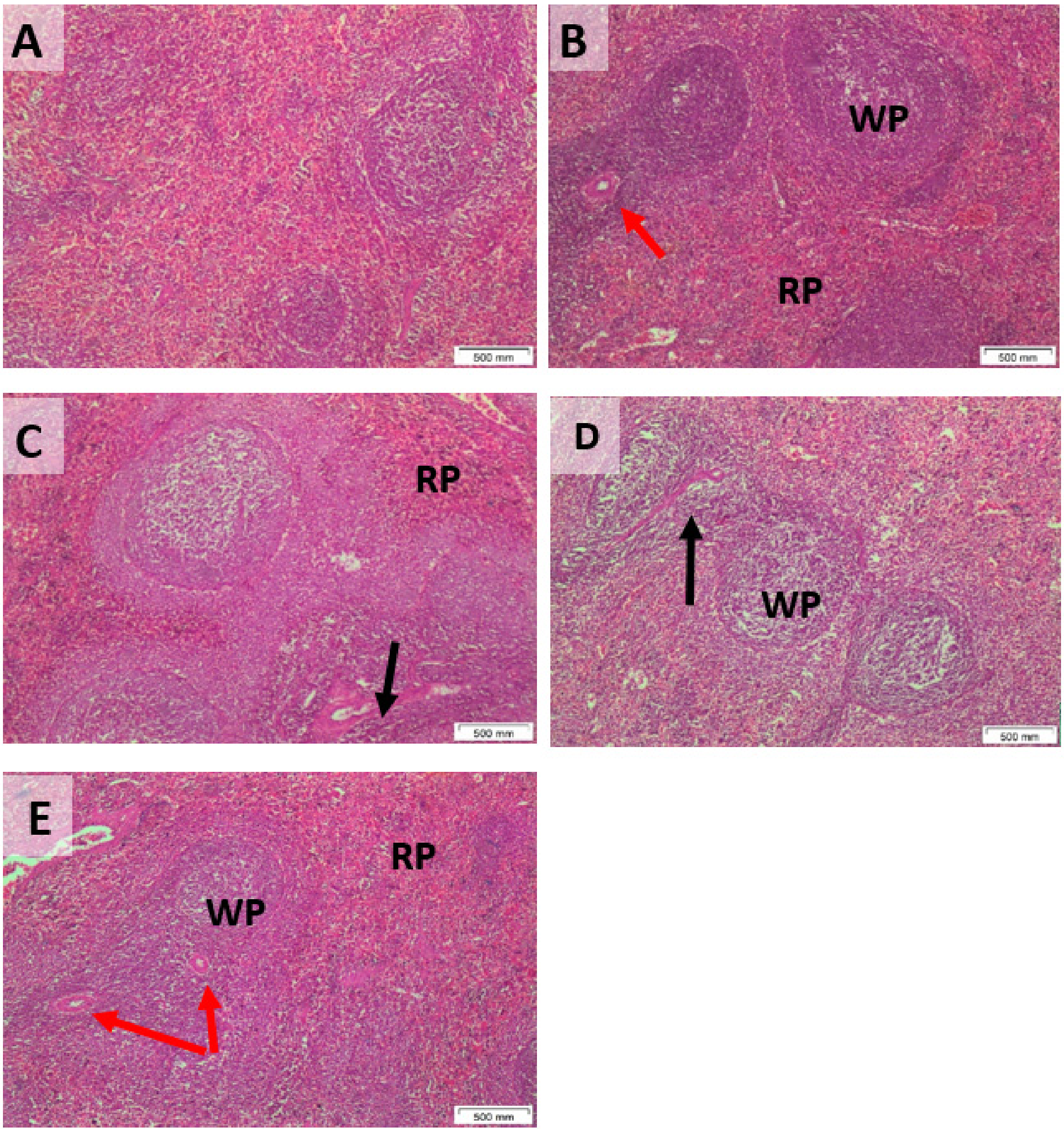

Figure S18. Photomicrography of Spleen tissue (Male) for different studied groups (H&E 50x), have been sacrificed after (8 weeks) from injection. A-: Control group: This is the normal appearance of the spleen. B, C, D & E–: Groups– treated with 3, 6, 15, & 30 µg respectively: have the same picture, showing white pulp (WP) surrounding a central arteriole (red arrow). The red pulp (RP) forms the bulk of the splenic parenchyma, normal appearance of trabeculae (black arrows).

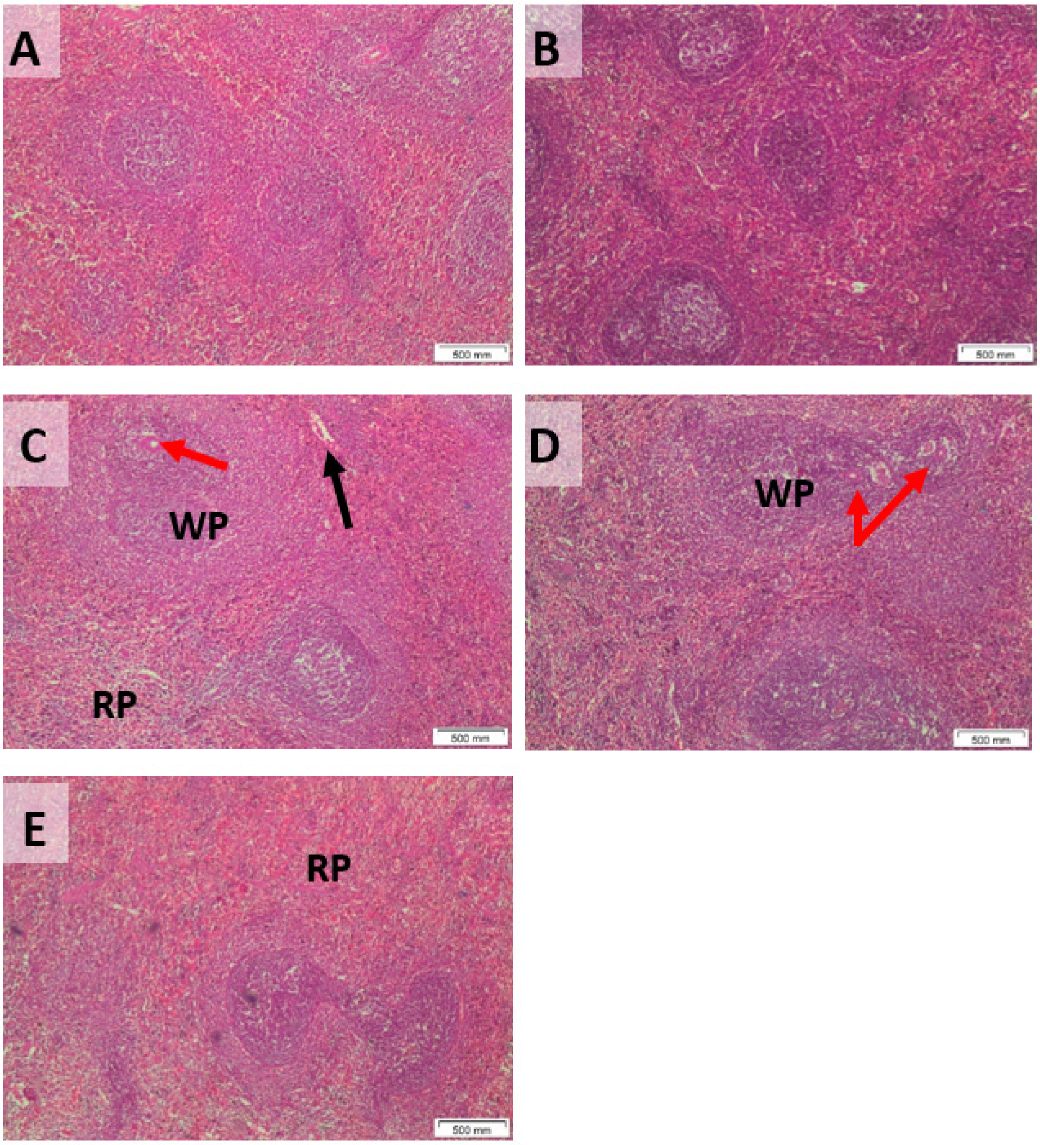

Figure S19. Photomicrography of Spleen tissue (Female) for different studied groups (H&E 50x), have been sacrificed after (8 weeks) from injection. A–: Control group: This is the normal appearance of the spleen. B,C, D & E–: Groups– treated with 3, 6, 15, & 30 μg respectively: have the same picture, showing white pulp (WP) surrounding a central arteriole (red arrow). The red pulp (RP) forms the bulk of the splenic parenchyma, normal appearance of trabeculae (black arrows).

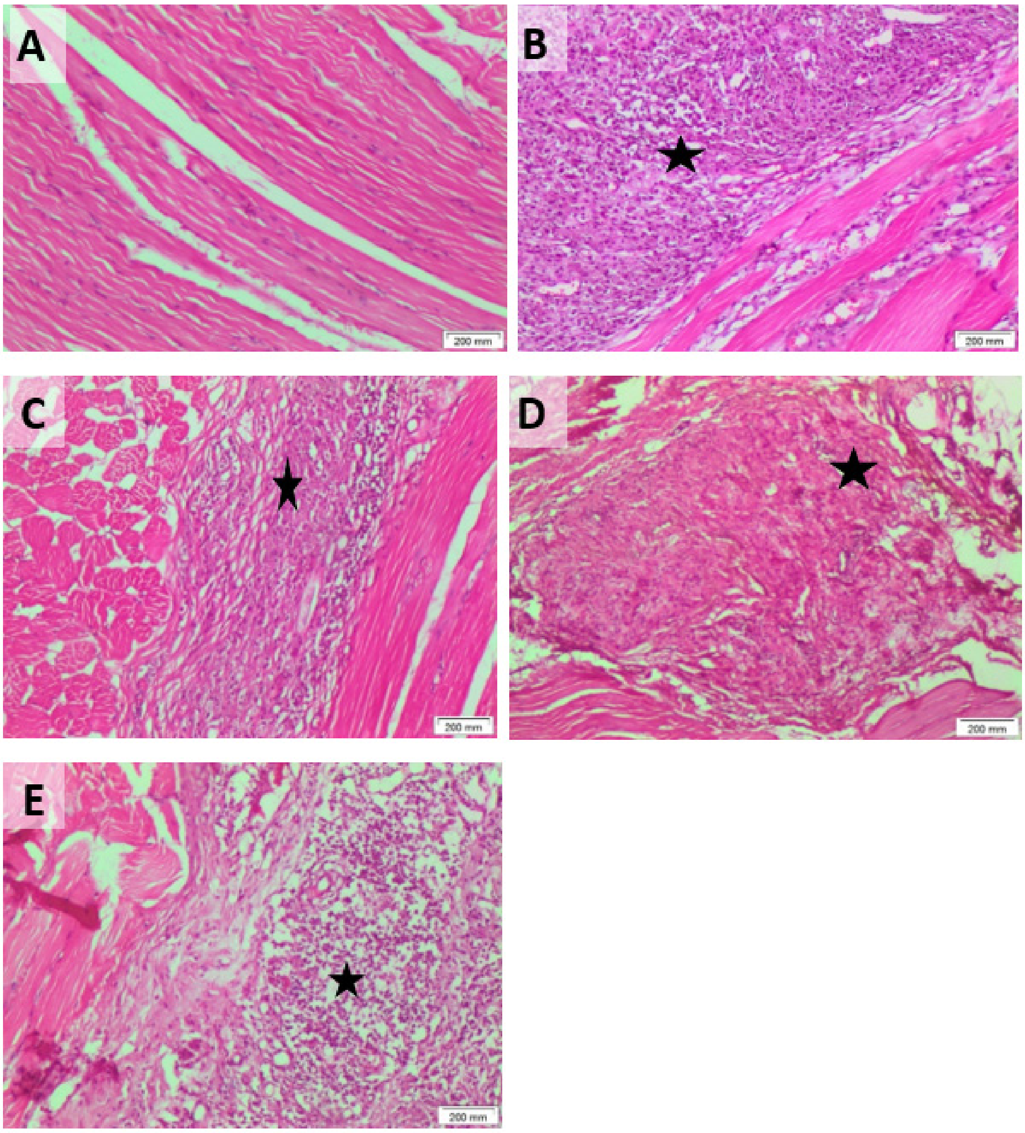

Figure S20. Photomicrography of Muscle tissue (site of injection) (Male) for different studied groups (H&E 100x), have been sacrificed after (8 weeks) from injection. A: –Control group: showing normal appearing muscle fascicles without any evidence of inflammatory reaction. B, C, D & E–: Groups– treated with 3, 6, 15, & 30 µg respectively : showing chronic inflammatory immune response (black stars) as the muscle fascicles disrupted by focal area formed predominantly of foamy histocytic aggregates with few polymorphic nuclear leukocytes (PNL).

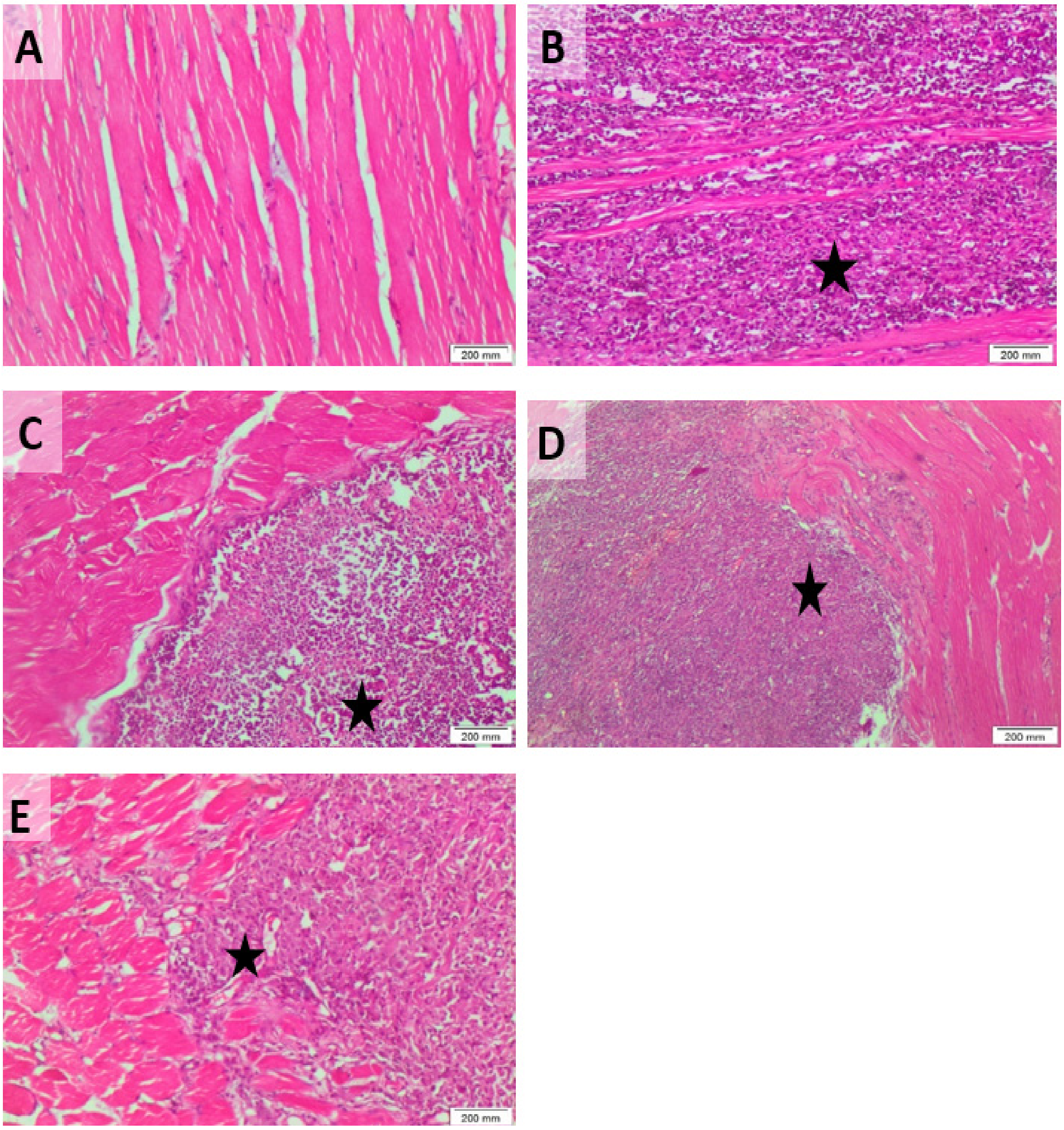

Figure S21. Photomicrography of Muscle tissue (site of injection) (Female) for different studied groups (H&E 100x), have been sacrificed after ~~(~~8 weeks~~)~~ from injection. A: ~~–~~Control group: showing normal appearing muscle fascicles without any evidence of inflammatory reaction. B, C, D & E~~–~~: Groups~~–~~ treated with 3, 6, 15, & 30 μg respectively: showing chronic inflammatory immune response (black stars) as the muscle fascicles disrupted by focal area formed predominantly of foamy histocytic aggregates with few polymorphic nuclear leukocytes (PNL).

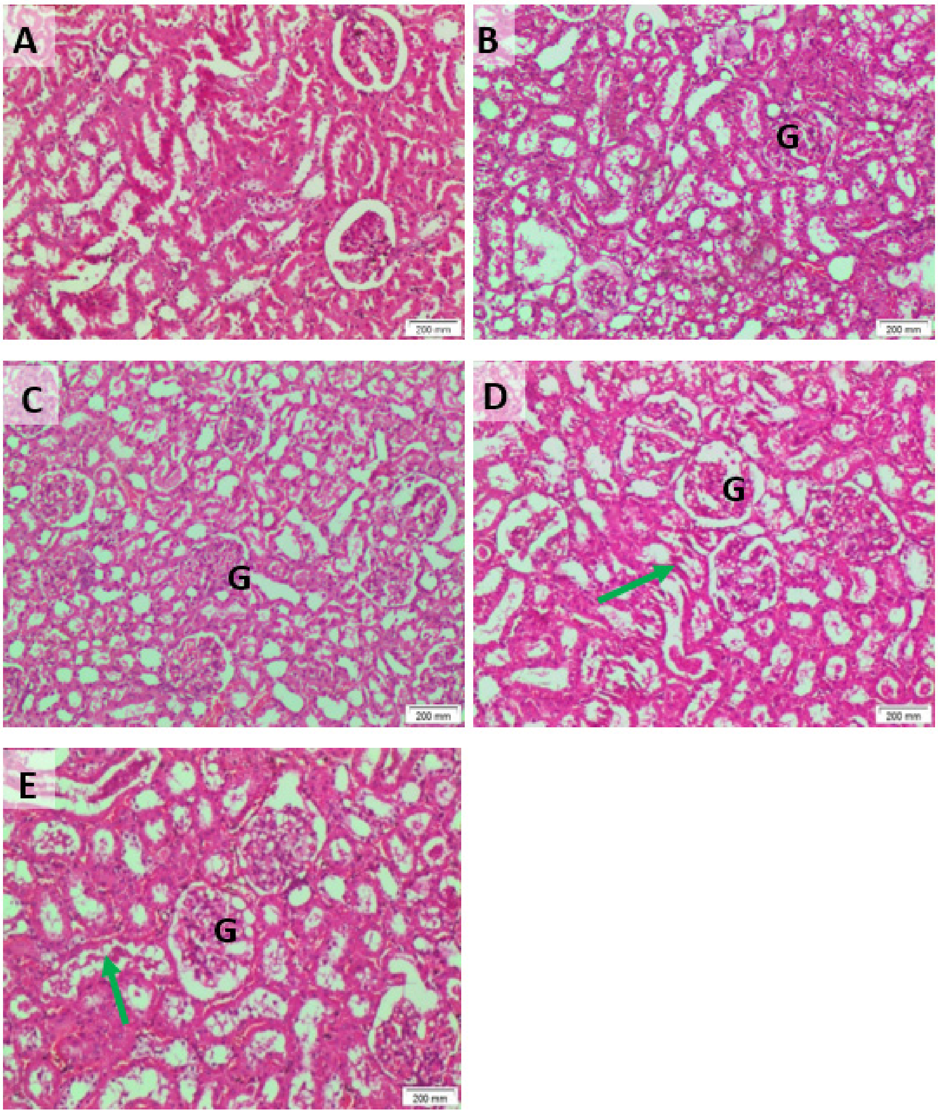

Figure S22. Photomicrography of Kidney tissue (Male), for different studied groups (H&E 100x), have been sacrificed after (8 weeks) from injection. A: Control group; showing normal renal architecture with well-formed glomeruli, average Bowman`s space and normal thickness of the tubules. B & C: Groups –treated with 3 & 6 µg respectively: have the same picture with normal glomeruli (G). D & E: Groups– treated with 15 & 30 µg respectively: have the same picture, normal glomeruli (G) with mild tublo-epithelial degeneration (green arrows).

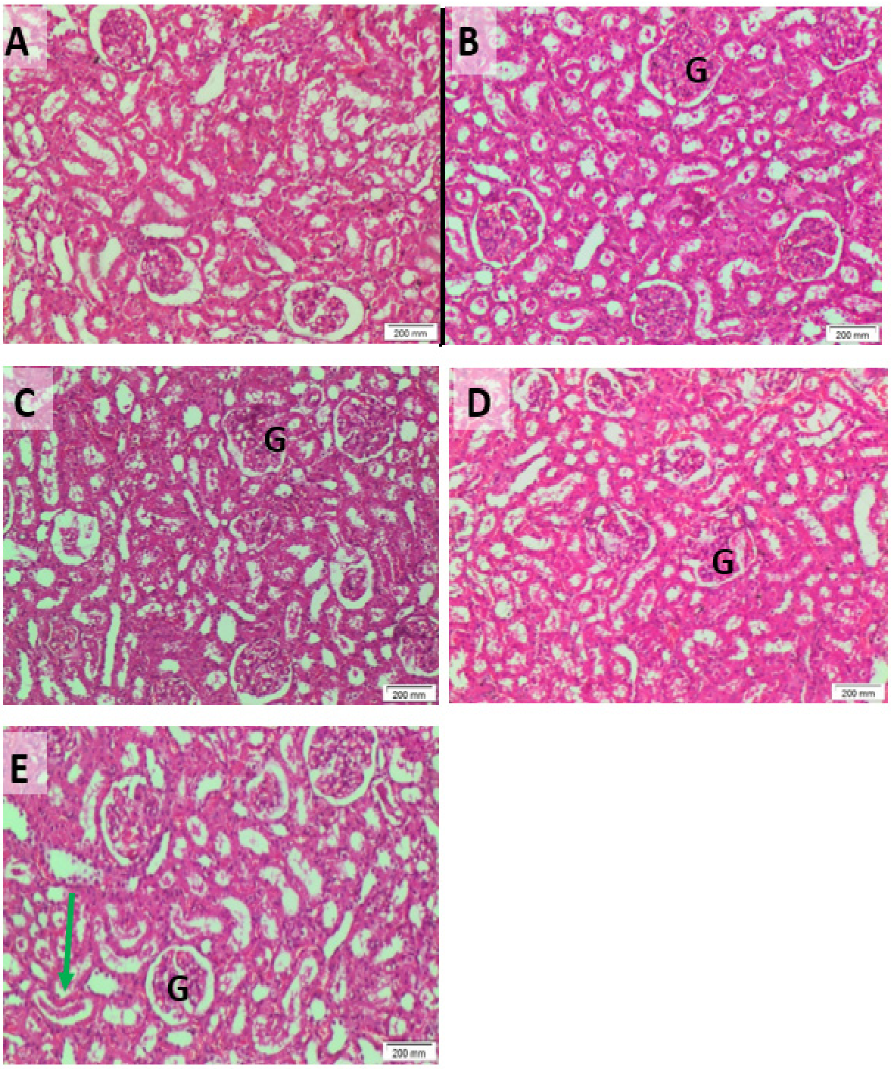

Figure S23. Photomicrography of Kidney tissue (Female), for different studied groups (H&E 100x), have been sacrificed after (8 weeks) from injection. A: Control group; showing normal renal architecture with well-formed glomeruli, average Bowman`s space and normal thickness of the tubules. B, C & D: Groups– treated with 3, 6, & 15 μg respectively: have the same picture with normal glomeruli (G). E: Groups— treated with 30 μg: have the same picture–, normal glomeruli (G) with mild tublo-epithelial degeneration (green arrows).

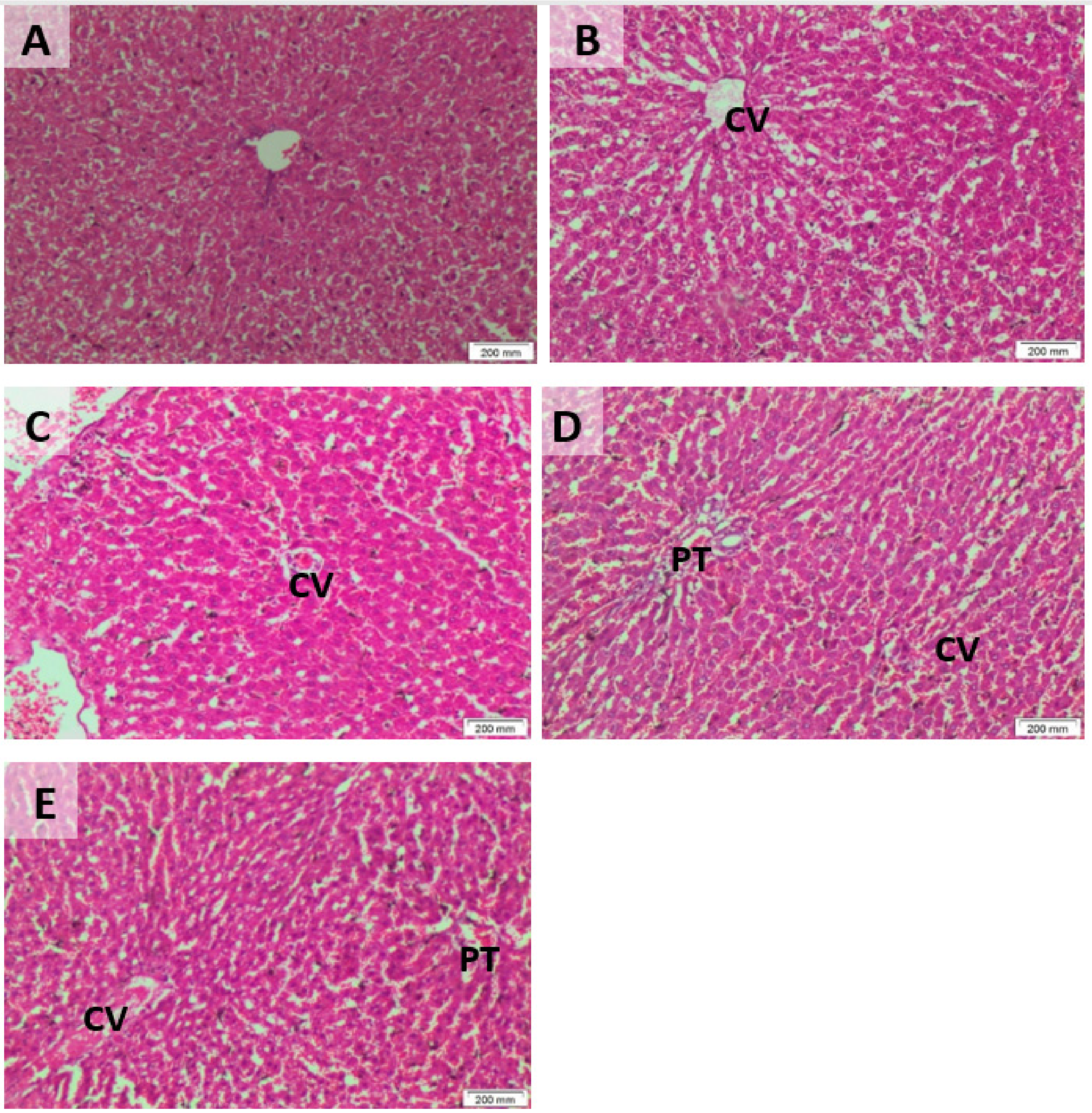

Figure S24. Photomicrography of Liver tissue (Male), for different studied groups (H&E 100x), have been sacrificed after (8 weeks) from injection. A: Control group; showing normal hepatic architecture composed of small lobules formed of hepatocytes which arranged as cords connecting the portal tracts (PT) in the periphery to the central veins (CV). B, C, D, E: Groups– treated with 3, 6, 15, & 30 μg respectively: have the same picture with mild congested dilated hepatic vessels.

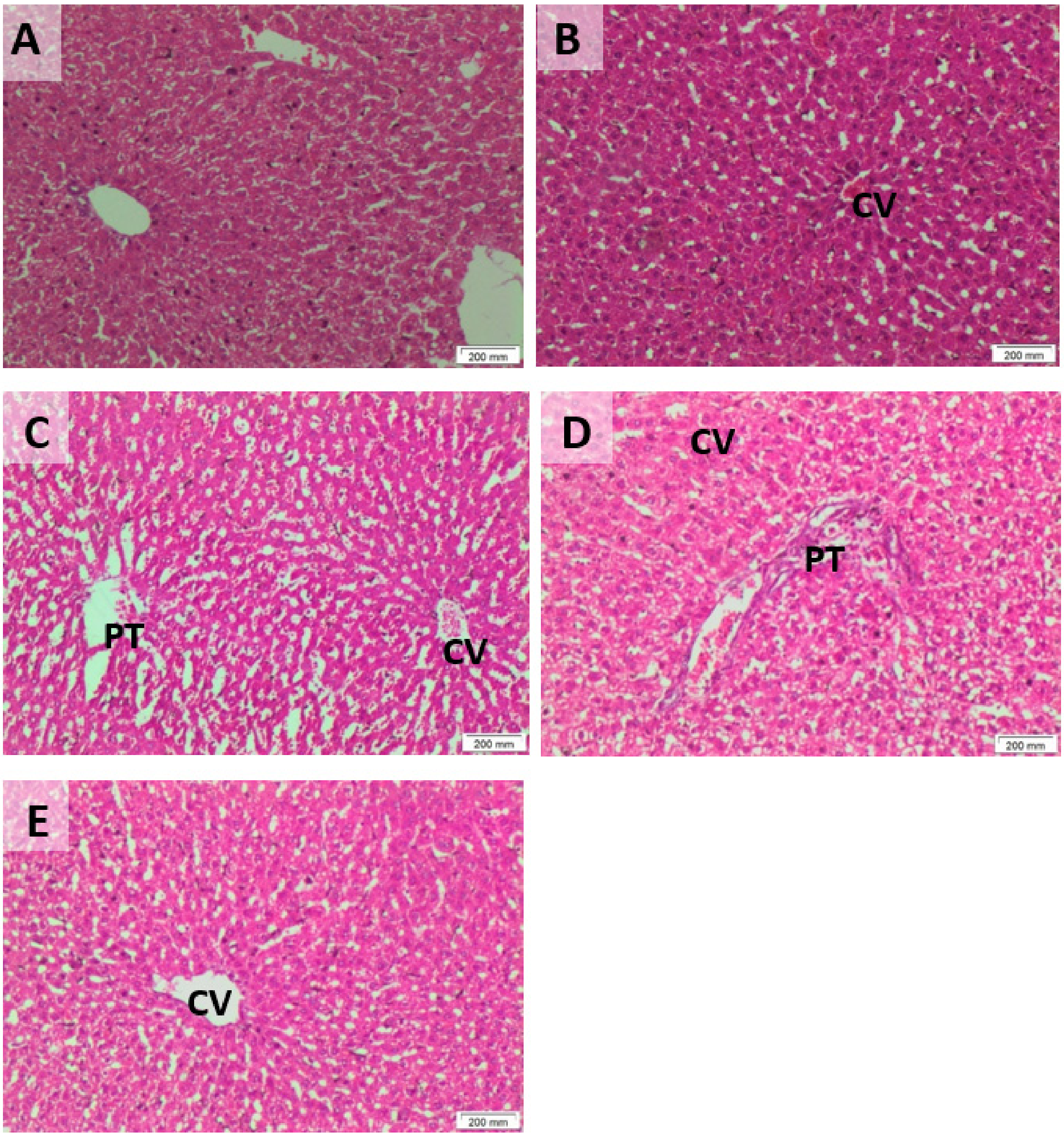

Figure S25. Photomicrography of Liver tissue (Female), for different studied groups (H&E 100x), have been sacrificed after (8 weeks) from injection. A: Control group; showing normal hepatic architecture composed of small lobules formed of hepatocytes which arranged as cords connecting the portal tracts (PT) in the periphery to the central veins (CV). B, C, D, E: Groups– treated with 3, 6, 15, & 30 µg respectively: have the same picture with mild congested dilated hepatic vessels.

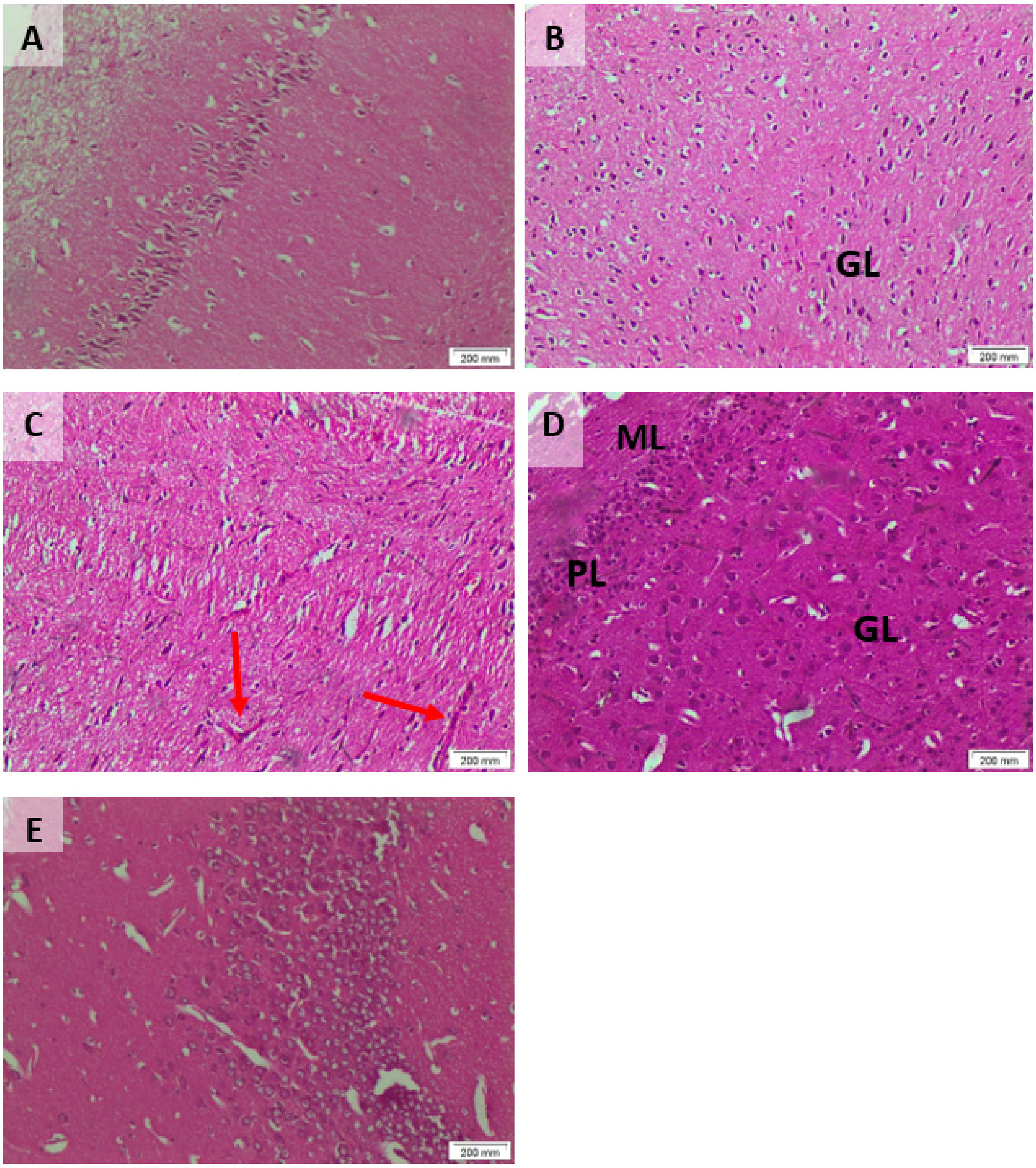

Figure S26. Photomicrography of Brain, for different studied groups (H&E 100x), have been sacrificed after ~~(~~8 weeks~~)~~ from injection. A: ~~, B, C, D, E;~~ Control group~~,~~. B, C, D, E: Groups treated with 3, 6, 15, & 30 µg respectively; Showing the cortex (gray matter) formed of outer molecular layer (ML), pyramidal cell layer with medium-sized pyramidal neurons (PL). Below this is the inner granular layer (GL) of larger pyramidal neurons, healthy neuronal vasculature (red arrows).

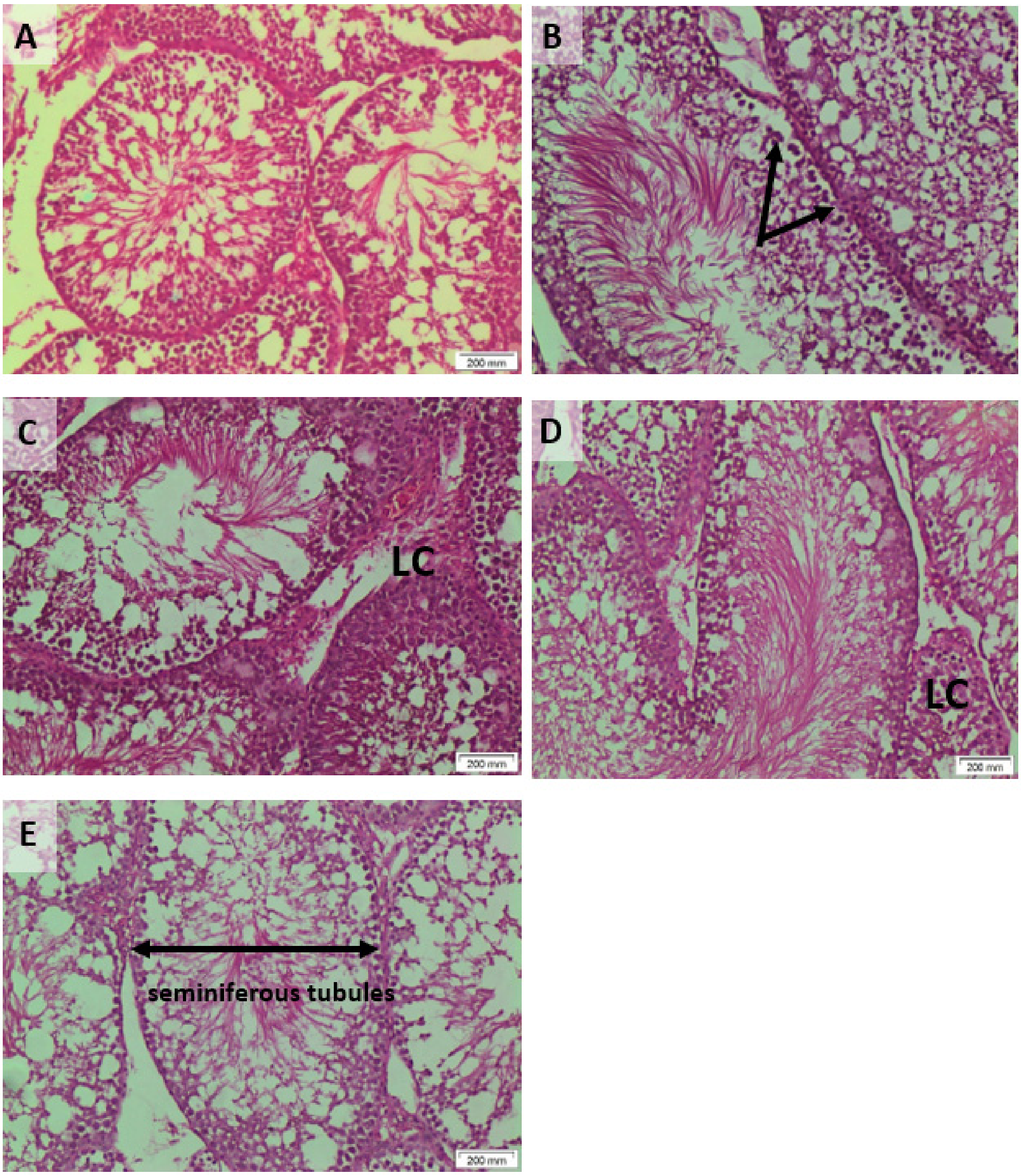

Figure S27. Photomicrography of Testes, for different studied groups (H&E 100x), have been sacrificed after (8 weeks) from injection. A, : Control group. B, C, D, E: ; Control group , Groups treated with 3, 6, 15, & 30 μg respectively; showing normal shape of seminiferous tubules (arrow double heads) with normal arrangement of spermatogonia and Sertoli cells (black arrows) resting on intact basement membrane separated by Leyding cells (LC).

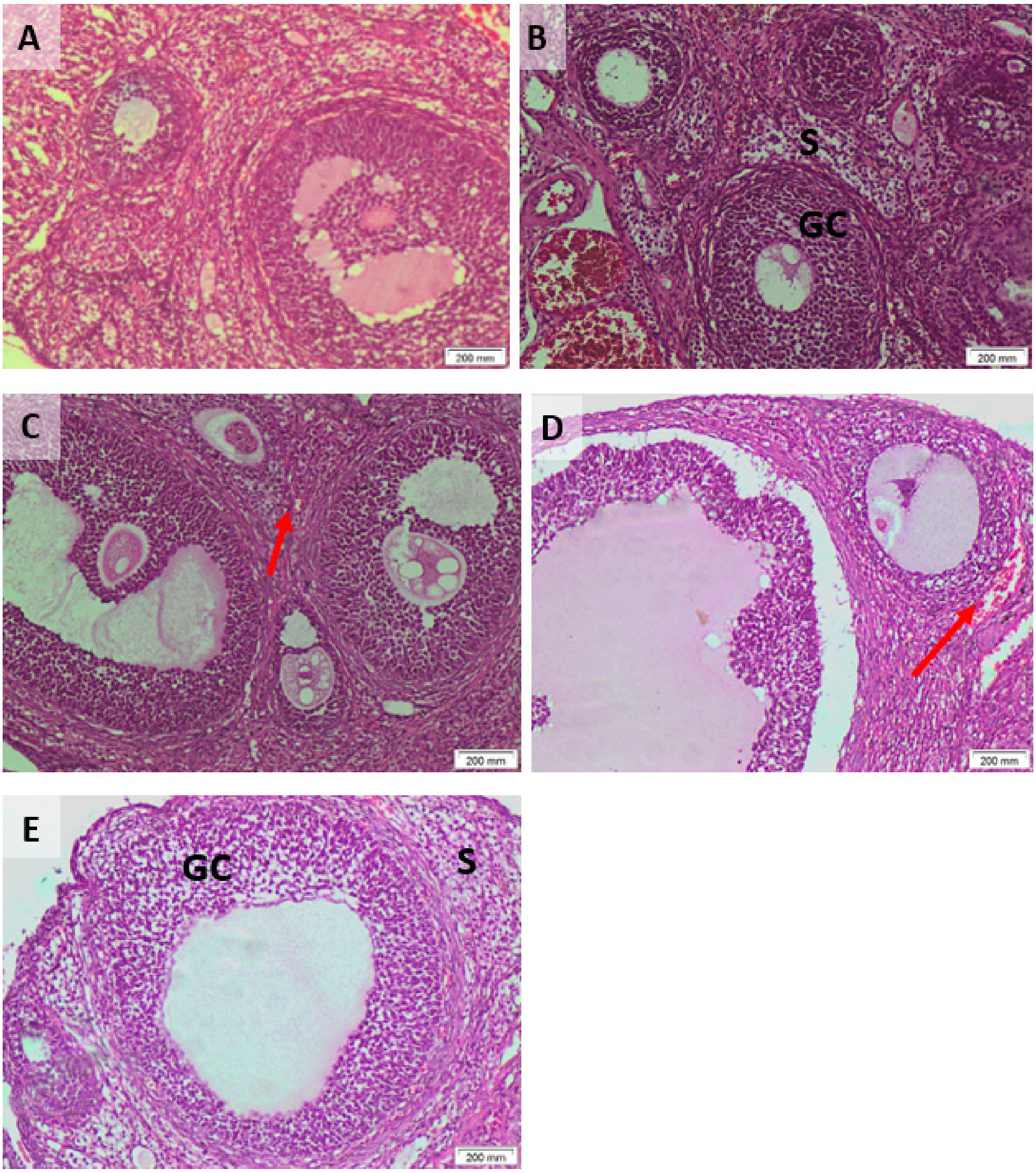

Figure S28. Photomicrography of Ovaries, for different studied groups (H&E 100x), have been sacrificed after ~~(~~8 weeks~~)~~ from injection.~~,~~ A~~, -~~: Control group. B, C, D, E:~~; Control group ,~~ Groups treated with 3, 6, 15, & 30 μg respectively; showing in both the presence of all types of follicles, normal vascularity (red arrows), compact stroma and intact germinal epithelium (GC), follicles in different stages of development, the connective tissue surrounding the follicles is called the 'stroma' (S).

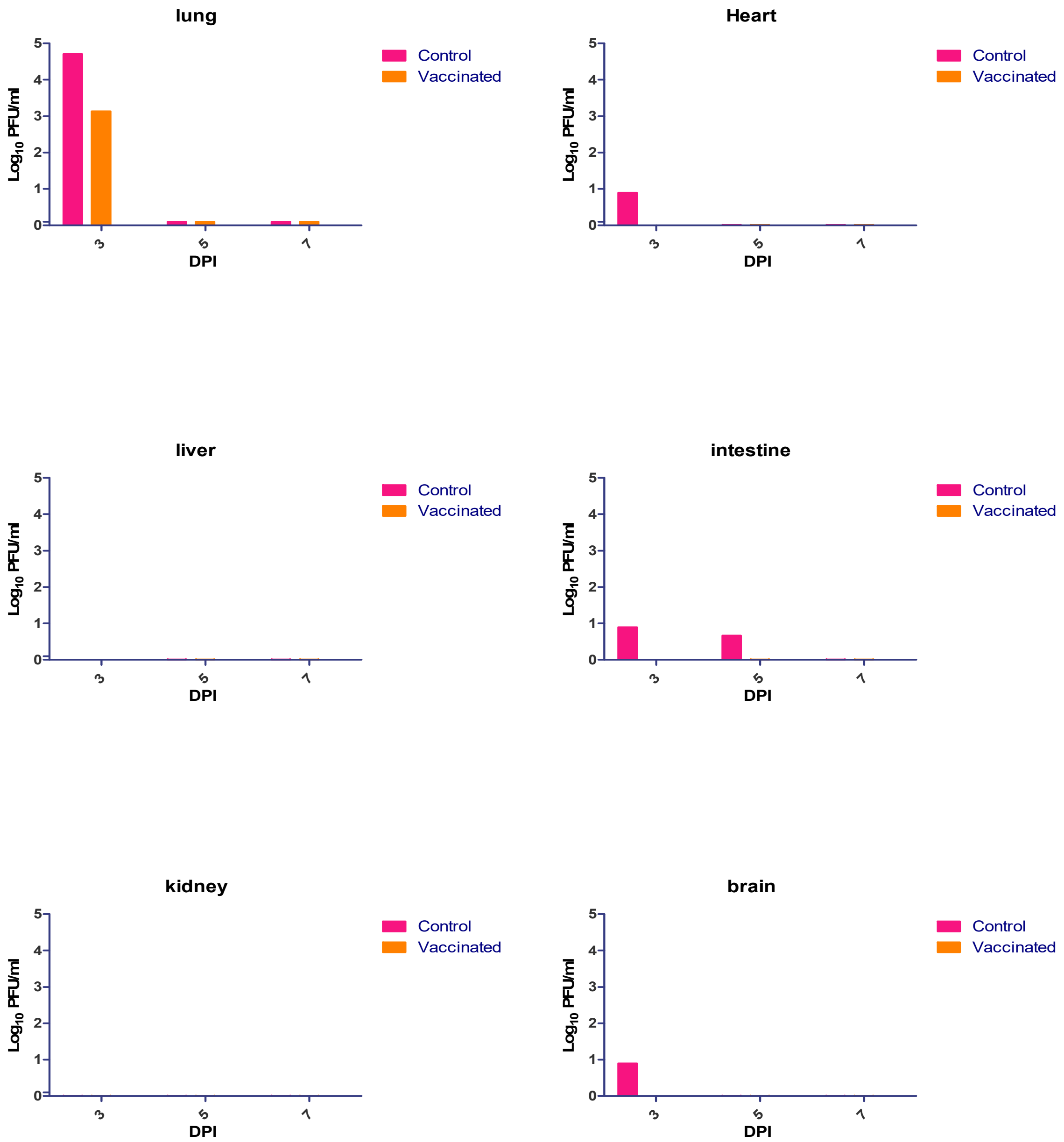

Figure S29. Viral titer of collected organs from vaccinated and control groups of infected hamsters. The viral titers were measured by plaque titration assay.

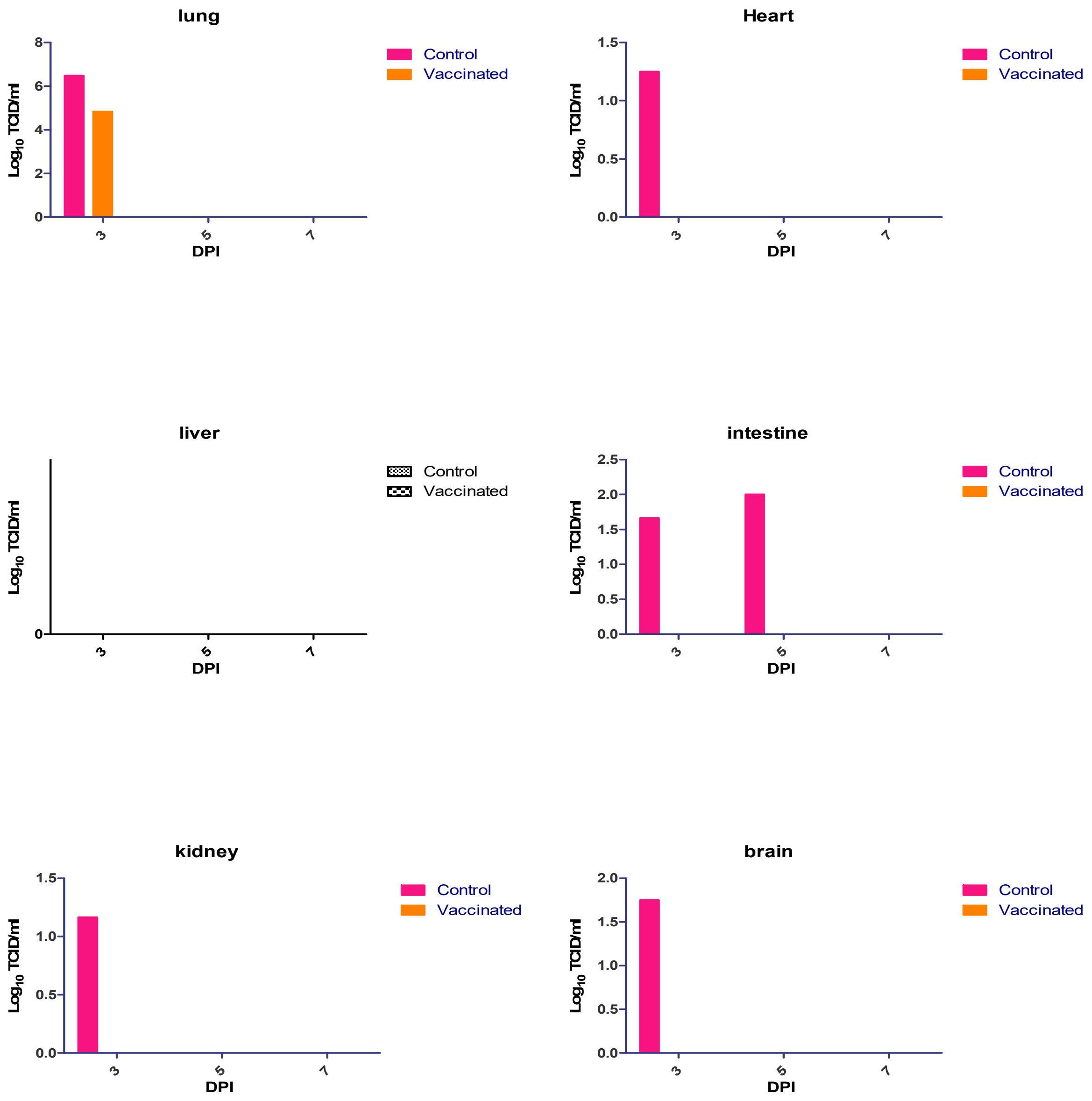

Figure S30. Viral titer of collected organs from vaccinated and control groups of infected hamsters. The viral titers were measured by TCID50.
